# Synthesis and preclinical evaluation of a novel 2-[^18^F]fluoropyridine-bearing rhodamine for myocardial perfusion imaging with PET

**DOI:** 10.1039/d6ra08884h

**Published:** 2026-11-05

**Authors:** Maya Mahmood, Emily Murrell, Seniha Ozyuncu, Junchao Tong, Angelina Lui, James A. H. Inkster

**Affiliations:** a McMaster University, Department of Chemistry and Chemical Biology Hamilton ON L8S 4M1 Canada inksterj@mcmaster.ca; b Brain Health Imaging Centre, Centre for Addiction and Mental Health Toronto ON M5T 1R8 Canada; c Department of Psychiatry, University of Toronto Toronto ON M5T1R8 Canada

## Abstract

[^18^F]Rho6G-DEG-F is a rhodamine-based PET radiotracer for myocardial perfusion imaging (MPI) that exhibits excellent first-pass myocardial extraction, persistent cardiac retention, and high heart : liver ratios in rats and pigs. However, in human volunteers, heart : liver contrast was suboptimal. The primary goals of this study were to design an [^18^F]Rho6G-DEG-F analogue of attenuated lipophilicity for radiolabelling *via* nucleophilic heteroaromatic ^18^F-fluorination ([^18^F]Rho6G-DEG-Py-F; [^18^F]6), and to evaluate [^18^F]6 in rats and mice. To that end, precursors bearing 2-nitro- (5) and 2-trimethylammonium triflate (8)- pyridine groups were prepared in overall yields of 48% and 29%, starting from rhodamine 6G; final purification did not require C18 chromatography. Manual standard radiofluorination conditions (TEA[^18^F]F/TEAHCO_3_/DMSO/160 °C/20 min) afforded a best decay-corrected radiochemical yield (DC-RCY) of 31% from 5 and 7% from 8. In the latter case, precursor and radiotracer were easily separated by HPLC (molar activity = 33 ± 8 GBq per µmol). Upon translation to an automated synthesizer, [^18^F]6 was obtained in DC-RCYs of 9.3% and 0.7% from 5 and 8 respectively; best molar activities were 50 and 144 GBq per µmol respectively. [^18^F]Rho6G-DEG-Py-F exhibited complete stability in rat and mouse serum over 120 min (37 °C). A radiometabolite assay in two rats showed differing metabolic profiles between sexes, but similar % intact tracer at 60 min (32–34%). Micro-PET imaging in rats demonstrated extended myocardial retention (SUV > 1.0 at 120 min), gradual liver washout, and high heart : liver ratios (3.7 in male, 2.5 in female at 120 min); however, similar experiments in mice revealed low heart uptake and rapid metabolism (SUV ∼ 0.3; heart : liver ratio = 1.0 at 120 min).

## Abstract 2

A [^18^F]fluoropyridine-bearing rhodamine has been designed for myocardial perfusion imaging. Straightforward synthesis of precursors and automated radiolabeling methods permitted the evaluation of [^18^F]Rho6G-DEG-Py-F uptake into rat hearts with PET.

## Graphical abstract

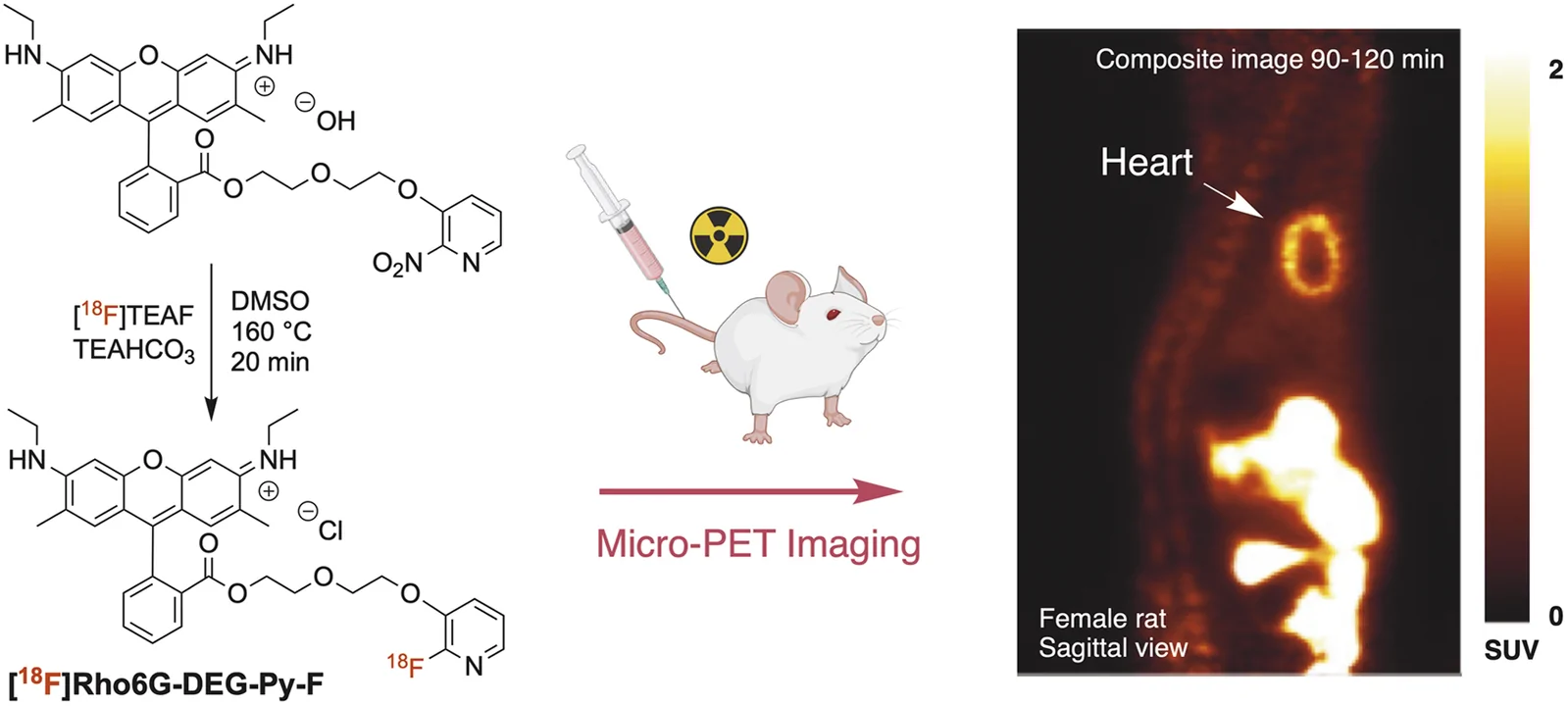

## Introduction

Myocardial perfusion imaging (MPI) is a well-validated molecular imaging technique that primarily uses single photon emission computed tomography (SPECT) radiopharmaceuticals for the non-invasive diagnosis and management of cardiovascular diseases associated with reduced myocardial blood flow. For decades, SPECT tracers such as ^99m^Tc-sestamibi and ^99m^Tc-tetrofosmin have been available for this application.^1^ In contrast, positron emission tomography (PET)-based MPI is far less developed, despite several advantages associated with this molecular imaging modality, including high spatial resolution images, accurate attenuation correction, low radiation dose using many PET isotopes, and ability to quantifiably measure tracer concentration in tissues in dynamic scans. ^18^F is considered the “best-in-class” PET isotope as it affords images with excellent resolution due to its low positron energy (*β*_avg_^+^ = 250 keV) and thus, short positron range (2.3 mm in water).^1,2^ Additionally, ^18^F is readily available by proton bombardment [^18^O(p,n)^18^F reaction] in high specific activities on medical cyclotrons, and with its intermediate half-life (*t*_1/2_ = 109.8 min), confers insignificant radiation to patients while being long enough to allow for multi-step radiosyntheses.^2^

Despite these advantages, most MPI-PET scans carried out in a clinical setting today employ the short-lived radioisotopes (*i.e.* [^82^Rb]RbCl, [^13^N]NH_3_, and [^15^O]H_2_O); all exhibit half-lives of <10 min, which greatly complicates radiopharmaceutical production, transport, and administration.^1^ Efforts in ^18^F-labelled MPI agent design have yielded a few promising tracers, all of which target the abundant number of mitochondria found in cardiomyocytes. All exhibit high and sustained uptake into cardiomyocytes in the first circulatory pass through the heart. Notably, a pyridaben derivative with high affinity for mitochondrial complex I ([^18^F]Flurpiridaz; Flyrcado™) was approved in September 2024 for diagnoses of cardiac ischemia and infarct in patients with known or suspected coronary artery disease (CAD).^3^ In addition, three classes of ^18^F-bearing lipophilic cations remain in preclinical development: (1) triphenylphosphonium cations;^4^ (2) acridinium orange derivatives;^5^ and (3) rhodamine dyes.^6^

Rhodamines are fluorescent xanthene derivatives that have been used for decades in numerous chemical and cellular assays.^7^ They exhibit an affinity for the negatively-charged outer membranes of mitochondria and many accumulate in mitochondria in proportion to mitochondrial membrane potential.^8^ This makes them useful as potentiometric dyes for the *in vitro* and *ex vivo* study of diseases associated with mitochondrial dysfunction or activity, including cancer, bacterial infection, and ischemia-reperfusion injury (IRI).^9^

Although the use of rhodamines as drug products and optical molecular imaging agents has been complicated by their capacity to cause cellular, genetic and organ-level damage,^10^ rhodamines labelled with ^18^F and administered at radiotracer concentrations have been evaluated as MPI-PET agents in a small number of pre-clinical imaging experiments. The Packard lab observed profound differences *in vivo* biodistribution depending on the xanthene analogue^6^ and ^18^F-bearing (alkoxy)alkyl tether^11^ employed. Early results suggested that a rhodamine B derivative with a 2-(2-[^18^F]fluoroethoxy)ethyl ester chain was superior to other ester tethers, with [^18^F]RhoB-DEG-F exhibiting increased cardiac uptake and greater stability in mouse serum when compared to a 2-(2-[^18^F]fluoro)ethyl ester-modified [^18^F]RhoB derivative (Fig. 1).^11,12^ In a later preclinical study, the rhodamine 19 [^18^F]fluoroethoxyethyl ester, [^18^F]Rho6G-DEG-F ([^18^F]1; Fig. 2A), was found to localize into rat cardiomyocytes in a sustained fashion (1.3% ± 0.5% ID per g at 15 min; 1.8% ± 0.2% ID per g at 120 min, *n* = 4).^6^ [^18^F]1 has since been evaluated as a potential MPI-PET agent in pigs,^13,14^ and first-in-human PET studies,^15^ as well as a means to monitor the *ex vivo* and *in vivo* distribution of radiolabelled mitochondria in PET experiments designed to evaluate mitochondrial transplantation therapy after IRI.^9,13,14,16^ Notably, [^18^F]1 exhibits a high first-pass myocardial extraction fraction in swine (79.4 ± 7.1%, *n* = 4)^17^ and correlates with tracer uptake over a wide range of blood flows;^18^ both of these characteristics are critical for successful MPI-PET. Also important is the ratio of tracer uptake into heart *vs.* liver, owing to the close proximity of these two organs in humans. The heart : liver (H : L) ratio of [^18^F]1 in rats at 55 min post-injection was 4.5, indicating good contrast between the two organs at this timepoint.^6^

**Fig. 1:**
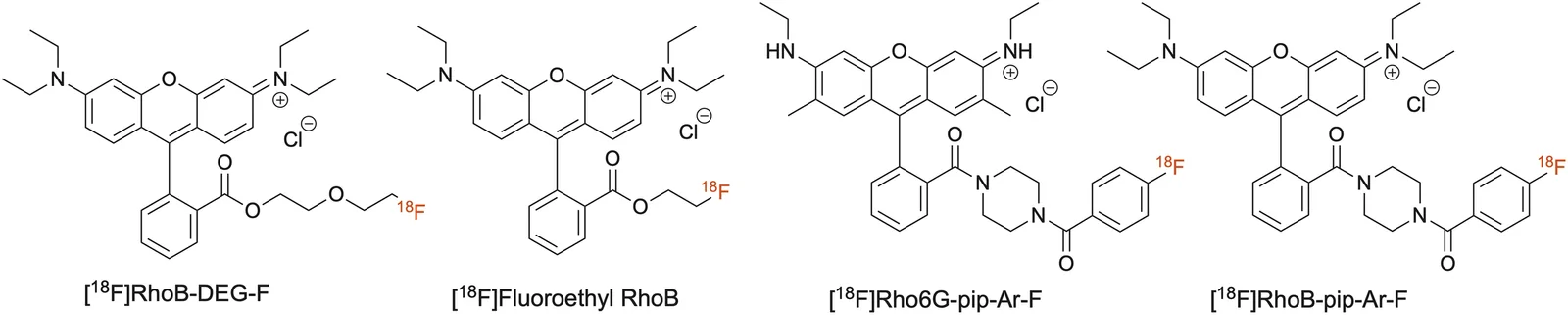
Structures of some previously reported ^18^F-rhodamine esters and amides.

**Fig. 2:**
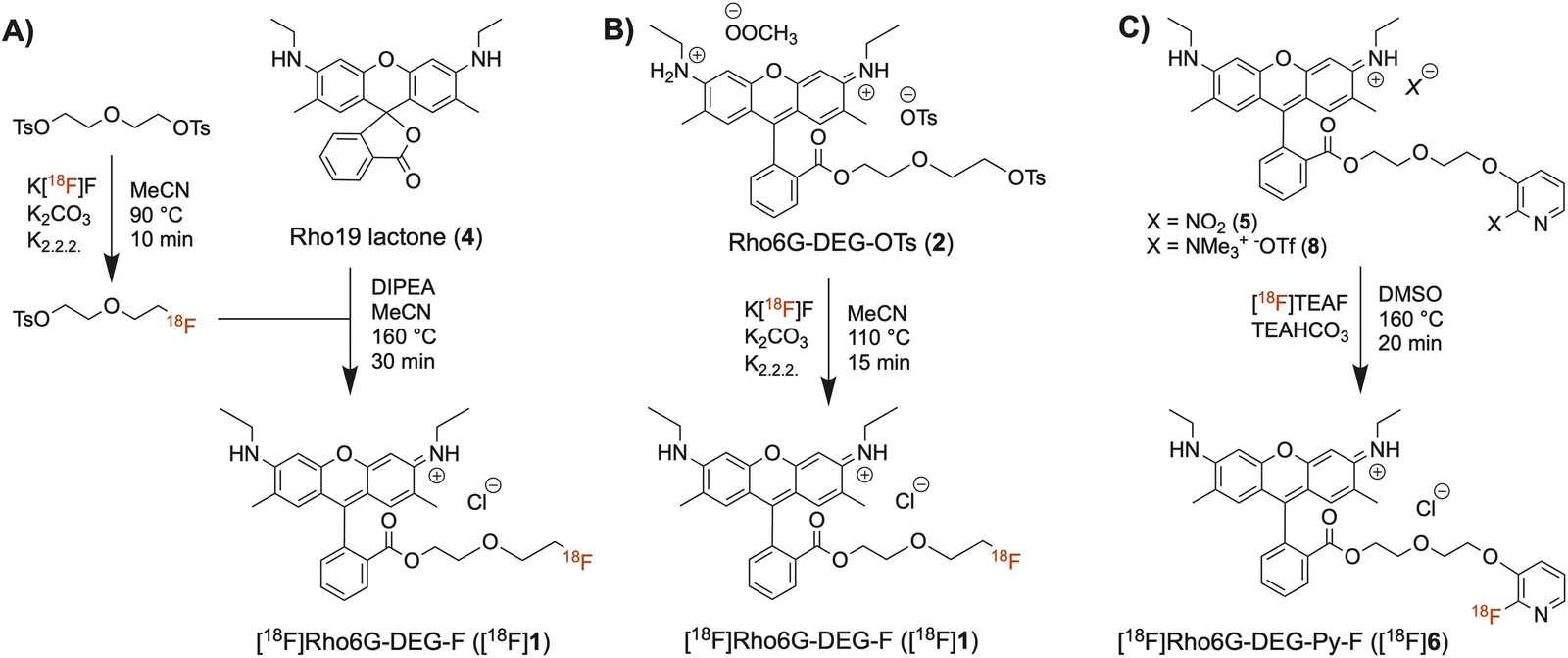
Synthesis of (A) [^18^F]Rho6G-DEG-F ([^18^F]1) *via* two-step method.^6^ Rhodamine 19 is added in its spirolactone form, which converts to the inner salt; (B) [^18^F]1*via* one-step nucleophilic aliphatic substitution;^18^ and (C) [^18^F]Rho6G-DEG-Py-F ([^18^F]6) *via* nucleophilic aromatic substitution (this work). Injected radiotracers exist in chloride form after formulation in isotonic saline.

[^18^F]1 was first synthesized indirectly through conventional nucleophilic aliphatic ^18^F-fluorination of bis-tosylated diethylene glycol, followed by conjugation of the corresponding ^18^F prosthetic group to rhodamine 19 (Fig. 2A).^6^ This manual protocol furnished tracer in quantities sufficient for preclinical µPET assay. However, the complexity of this 2-radiochemical-step ^18^F-labelling method, and the low molar activities of [^18^F]1 obtained (0.32 GBq per µmol) made it unsuitable for clinical translation. Thus, a direct radiolabelling strategy was developed (Fig. 2B), starting from tosylated precursor Rho6G-DEG-OTs (2). This approach was translated to an automated synthesis unit (ASU), which allowed for the sterile preparation of [^18^F]1 and first-in-human PET scans.^19^ Outcomes of these various radiosynthetic protocols can be found in Table 3.

Many of the challenges associated with the preclinical assessment and clinical translation of [^18^F]1 pertained to the preparation of precursor 2. As delocalized lipophilic cations, rhodamines can be purified on silica gel using methylene chloride and methanol as eluent matrix. However, rhodamine cations exhibit high affinity for chloride counterions, and even the low concentrations of HCl found in methylene chloride result in samples of 2 that ^18^F-label poorly due to Cl^−^/[^18^F]F^−^ competition. Thus, preparative C18 flash chromatography was required for final purification on a 0.5–1 g scale.^19^ Another drawback of 2 is its limited shelf-stability, even when kept under inert gas and refrigerated. Apart from negatively affecting radiochemical yields (RCYs), de-tosylated rhodamine alcohol impurity is difficult to separate from [^18^F]1 by high-performance liquid chromatography (HPLC), to the detriment of final molar activities (*A*_m_).

Synthetic considerations aside, [^18^F]1 unexpectedly exhibited sustained uptake into liver in a Phase 0 trial of four healthy volunteers.^15^ In an effort to identify ^18^F-rhodamine MPI agents with improved H : L ratios at early timepoints, investigators prepared derivatives that were predicted to be less susceptible to both defluorination and ester hydrolysis. Thus, [^18^F]Rho6G-pip-Ar-F and related rhodamine B derivative [^18^F]RhoB-pip-Ar-F each possess a tertiary amide and [^18^F]fluorophenyl group (Fig. 1), as compared to [^18^F]1's ester and ^18^F-diethylene glycol moieties respectively. Unfortunately, these rhodamine amides exhibited reduced uptake in the heart and slow clearance from the liver (H : L ratio = 0.14–0.20 at 60 min).^20^ It is not yet clear which one, or both, of the new functionalities (*i.e.* amide or [^18^F]fluoroarene) contributed to the observed suboptimal biodistribution patterns.

Our labs have launched an effort to design alternatives to [^18^F]1 that are easier to synthesize and exhibit comparatively equal or better MPI-PET characteristics. On this basis, our ‘next generation’ MPI tracer is envisioned to include a robust 2-[^18^F]fluoropyridine moiety, while retaining the diethylene glycol ester chain ([^18^F]Rho6G-DEG-Py-F; [^18^F]6). Radiofluorination occurs *via* nucleophilic aromatic substitution at a nitro or trimethylammonium group at the 2-position of the pyridine ring (Fig. 2C), based on previous studies showing optimal [^18^F]F^−^ incorporation at this position in comparison to *meta*- and *para*- substitution.^21^ This manuscript describes the development of [^18^F]6 as a candidate ^18^F-PET-MPI agent, including non-radioactive synthesis of precursors and ^19^F standard, optimization of manual and automated radiolabelling conditions, and preclinical PET imaging in rats and mice.

## Results and discussion

### Synthesis of [^18^F]Rho6G-DEG-Py-F precursors and standard

Precursors Rho6G-DEG-Py-NO_2_ (5) and Rho6G-DEG-Py-NMe_3_^+−^OTf (8), along with ^19^F standard 6, were prepared in a manner similar to the previously reported synthesis of precursor 2, starting from inexpensive and widely available rhodamine 6G chloride (3; Fig. 3).^19^ The chloride counterion was removed *via* pyrolysis of 3 (ref. 7) at 250 °C to form the neutral rhodamine 19 lactone (4). Transesterification of 4 with 2-(2-((2-nitropyridin-3-yl)oxy)ethoxy)ethyl tosylate (9), 2-(2-((2-dimethylaminopyridin-3-yl)oxy)ethoxy)ethyl tosylate (10), or 2-(2-((2-fluoropyridin-3-yl)oxy)ethoxy)ethyl tosylate (11) was achieved in refluxing acetonitrile (MeCN) in the presence of *N*,*N*-diisopropylethylamine (DIPEA) base. Synthetic details for these bifunctional chains can be found in the supporting information (SI) document. Nucleophilic substitution to form rhodamine esters 5, 6, and 7 proceeded through the ring-opened form of rhodamine 19 lactone (*i.e.* rhodamine 19 inner salt), and takes advantage of the unusual nucleophilic character of 2′-carboxyl-substituted rhodamine dyes.^22^ The extent of transesterification was monitored by C18 HPLC (HPLC A; Program E). As initially reported for the synthesis of 2, the transesterification reactions did not proceed to completion until reaction mixtures were concentrated to dryness at 130 °C.^19^

**Fig. 3:**
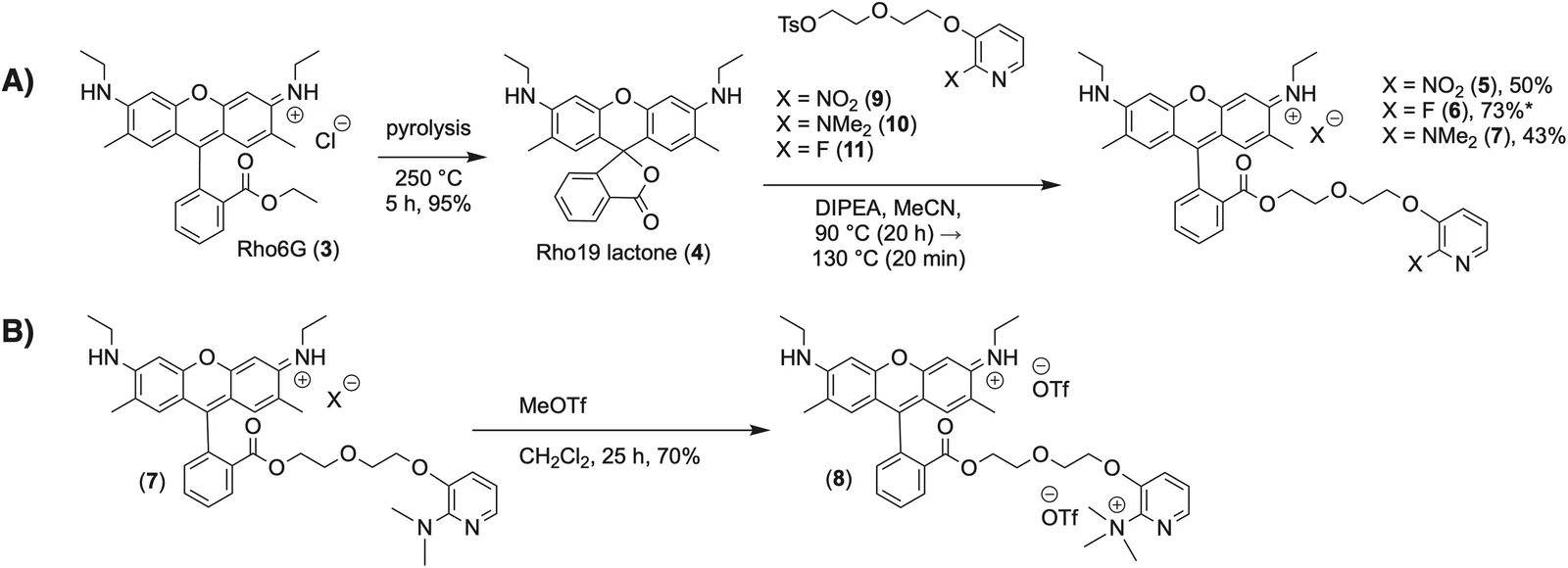
Synthesis schemes for (A) [^18^F]Rho6G-DEG-Py-F precursor 5, ^19^F standard 6 and compound 7, and (B) [^18^F]Rho6G-DEG-Py-F precursor 8. For compounds 5, 6, and 7, counterions (X^−^) are assumed to be ^−^OH. For compound 8, counterion = ^−^OTf. *93–95% pure.

Notably, rhodamine esters 5, 6, and 7 did not require preparative C18 chromatography for purification but were instead isolated on gram scale using flash columns of neutral alumina, using 9 : 1 ethyl acetate–methanol as eluent. In addition to being cost effective, this purification strategy does not require chlorinated solvents. Tosylate counterion was not observed in the ^1^H & ^13^C NMR of 5, 6, and 7 after purification on alumina sorbent. This is in contrast with C18-based purification of Rho6G-DEG-OTs (2), in which 2 was recovered as a tosylate/formate double salt (Fig. 2A).^19^ Product specifications for the alumina used for the isolation of 5, 6 and 7 (Sigma-Aldrich; Prod. # 199974) cite the largest impurity assayed as Na_2_O (<0.4%), which in conjunction with the sorbent's 5% water content (Brockmann I), should provide a significant source of hydroxide anions. This basic species can undergo rapid exchange with trace water, acid or intermolecular protons, and thus may not be detected by ^1^H-NMR.^23^

### Optimization of radio-HPLC conditions

To ensure that [^18^F]6 could be separated from precursor 5 following radiosynthesis, HPLC conditions allowing for separation of 5 and 6 were tested on an analytical C18 pentafluorophenyl (PFP, aka F5) column (Fig. S2 in the SI). Peak resolution (*R*) of > 1.5 was considered adequate for baseline separation of symmetrical peaks, while resolutions of > 2.0 were needed for tailing peaks. Although gradient programs provided better peak shapes, *R* under these conditions was < 1.5 (HPLC A, Program G). An isocratic program (HPLC A, Program I) afforded a trace with acceptable peak shape and resolution (*R* = 2.98), which informed the choice of a similar program for semi-preparative purification of [^18^F]6 (HPLC A, Program A, *i.e.* 50% MeCN in H_2_O with 0.1% TFA; *R* = 2.09).

### Manual radiosynthesis of [^18^F]Rho6G-DEG-Py-F ([^18^F]6)

In a bid to optimize the radiochemical yield of [^18^F]6, a series of radiolabelling experiments were conducted, varying base, phase transfer catalyst (PTC), and temperature (Table 1). Run 1 followed conditions standard for the synthesis of 2-[^18^F]fluoropyridines *via* nucleophilic aromatic substitution.^24^ Precursor 5 was heated (130 °C) in anhydrous DMSO in the presence of dried [^18^F]KF/K_2.2.2_ complex and K_2_CO_3_; this resulted in a low radiochemical conversion (12% RCC). When the temperature was increased to 160 °C, an improved RCC was observed (37%, an average of four separate radiolabellings; runs 2–5). Increasing the amount of precursor from 0.5 mg to 1 mg under these conditions did not significantly improve RCCs (41%; Run 6) and furthermore complicated the isolation of [^18^F]6 from the reaction matrix by radio-HPLC. Radio-HPLC analysis of the crude reaction mixtures revealed significant hydrolysis of precursor, which could be partially mitigated by using a milder base (tetraethylammonium bicarbonate, TEAHCO_3_), as shown in Fig. S1 in the SI. Overall, the highest RCC of [^18^F]6 from precursor 5 obtained was 72% (average across three experiments), using 7.2 µmol (1.38 mg) TEAHCO_3_ and 160 °C (runs 9–11; Fig. 2B). Reducing the temperature back to 130 °C resulted in a significant reduction in RCC (5%; Run 7), while halving the amount of base yielded a small reduction (55%; Run 8).

**Table 1:** Manual radiosyntheses of [^18^F]Rho6G-DEG-Py-F ([^18^F]6) from Rho6G-DEG-Py-NO_2_ (5) and Rho6G-DEG-Py-NMe_3_^+−^OTf (8)

Run^a^	Precursor	Base (µmol)	PTC (µmol)	Temp (°C)	%RCC ± σ^b^
1	5	K_2_CO_3_ (3.6)	K_2.2.2_ (9.6)	130	12 ± <1 (*n* = 3)
2–5	5	K_2_CO_3_ (3.6)	K_2.2.2_ (9.6)	160	37 ± 1 (*n* = 12)^c^
6^d^	5	K_2_CO_3_ (3.6)	K_2.2.2_ (9.6)	160	41 ± 6 (*n* = 3)
7	5	TEAHCO_3_ (7.2)		130	5 ± <1 (*n* = 3)
8	5	TEAHCO_3_ (3.6)		160	55 ± 7 (*n* = 3)
9–11	5	TEAHCO_3_ (7.2)		160	72 ± 4 (*n* = 9)^e^
12	8	K_2_CO_3_ (3.6)	K_2.2.2_ (9.6)	160	6 ± 1 (*n* = 3)
13	8	K_2_CO_3_ (3.6)	K_2.2.2_ (9.6)	130	4 ± <1 (*n* = 3)
14–21	8	TEAHCO_3_ (7.2)		160	3–25^f^

a Reaction conditions: 0.5 mg precursor, unless noted; 0.5 mL anhydrous DMSO; 20 min reaction time.

b Radiochemical conversion (RCC) calculated by radio-TLC.

c Average %RCC across four separate radio-experiments.

d 1 mg (1.25 µmol) of precursor 5 used.

e Average RCC across three separate radio-experiments.

f %RCC of runs 14–21 were estimated by HPLC and found to be 25, 12, 6, 3, 6, 4, 3, and 6%, in the order of performed experiments.

After semi-preparative HPLC purification (HPLC A, Program B), [^18^F]6 was isolated from the HPLC solvent by solid-phase extraction using a tC18 cartridge (Waters™), concentrated to dryness, and formulated in 10% EtOH in saline. Manual radiochemical yields (RCYs) of [^18^F]6 from start-of-synthesis (SOS) were 3–31% (*n* = 9) decay-corrected (DC). Fastest manual synthesis time was 162 min from SOS. This is comparable to manual RCYs of [^18^F]1 by Inkster *et al.* [Table 3, row 8 *vs.* row 2].^19^ Starting from precursor 5, molar activities of [^18^F]6 were higher than those found for [^18^F]1,^19^ but exhibited batch-to-batch variability (1–50 or 17 ± 19 GBq per µmol). This is due to a reduction in HPLC resolution observed during radiolabelling reactions *vs.* non-radioactive test separations (*vide supra*), as the result of injection matrices containing 0.5 mg of precursor, large injection volumes (1.5 mL), and a significant percentage of polar organic solvent (2 : 1 0.1% TFA_(aq)_ : DMSO).

Challenges associated with the chromatographic purification of [^18^F]6 prompted the synthesis and evaluation of precursor Rho6G-DEG-Py-NMe_3_^+−^OTf (8), which exhibits much shorter retention times than [^18^F]6 on C18 HPLC, resulting in a simpler collection step and formulations of higher *A*_m_. Manual radiolabeling of 8 proceeded in a manner similar to ^18^F-fluorinations with 5 (Table 1). Use of K_2.2.2_/K[^18^F]F/K_2_CO_3_ at both 130 °C and 160 °C for 20 min resulted in low RCCs by radio-TLC (runs 12 & 13). Using TEAHCO_3_ at 160 °C initially resulted in a RCC of 25%, which dropped to 12%, and then 4% in subsequent radiolabelling experiments (runs 14–21). The precursor batch showed no signs of degradation when re-assayed by HPLC and ^1^H-NMR; however, the compound gradually became deliquescent upon repeated exposure to air. Attempts to lyophilize 8 or dry under high vacuum overnight did not result in ^18^F-labellings of improved RCC. Nevertheless, as expected, purification of [^18^F]6 from doubly charged precursor 8 by semi-preparative HPLC was much more straightforward than purification from 5 (HPLC A, Program A, *t*_R,RAD_ = 17.41 min, *t*_R,530nm_ = 4.79 min), and final formulations were obtained in molar activity of 23–41 GBq per µmol (*n* = 6) at end-of-synthesis (EOS; Table 3, row 9). Manual radiochemical yields of [^18^F]6 from SOS were 1–7% DC (*n* = 6). Fastest manual synthesis time was 132 min from SOS.

### Automated radiosynthesis of [^18^F]Rho6G-DEG-Py-F ([^18^F]6)

The radiosynthesis of [^18^F]6 was translated to an automated synthesis module (GE TRACERlab FX2 N) using conditions similar to those employed for manual syntheses. Starting from nitro-bearing precursor 5, DC-RCYs of 8, 6, and 9% were achieved, starting from 22.2–33.3 GBq of [^18^F]F^−^ (600–900 mCi). These yields are comparable to those previously reported for [^18^F]1 using a GE Tracerlab FXF N automated synthesizer (Table 3, row 10 *vs.* row 3).^19^ From precursor 5, 962, 999 and 1887 MBq (26, 27, and 51 mCi) batches of [^18^F]6 were prepared; molar activities were estimated to be 39, 88 and 50 GBq per µmol at EOS, respectively, based on a mass standard curve of 6. The fastest automated synthesis time was 78 min from SOS. In two automated radio-experiments using trimethylammonium-bearing precursor 8, [^18^F]6 was prepared in low RCY (0.7% DC in both runs), resulting in 96.2 and 140.6 MBq (2.6 and 3.8 mCi) of [^18^F]6 at EOS with molar activities of 85 and 144 GBq per µmol, respectively. The fastest overall automated synthesis time was 67 min from SOS. Considering the suboptimal activity yields obtained using precursor 8, precursor 5 was chosen for the automated production of [^18^F]6 for use in subsequent preclinical imaging and radio-metabolite experiments.

From a radiosynthetic perspective, [^18^F]1 has been prepared in higher automated yields than [^18^F]6 (from 8) and in higher *A*_m_s than [^18^F]6 (from 5) (Table 3, row 3 *vs.* rows 10 & 11). However, it should be noted that the automated synthesis of [^18^F]1 was carried out with 3.7–5.6× the starting radioactivity as the experiments reported here (124.5 GBq *vs.* 22.2–33.3 GBq).^19^ This indicates that automated syntheses using nitro precursor 5 could easily provide radiopharmaceutical doses of [^18^F]6 sufficient for human PET imaging if appropriately scaled.

### Stability of [^18^F]Rho6G-DEG-Py-F in serum and plasma

[^18^F]6 demonstrated excellent stability in PBS (1×, pH 7.4), rat serum and mouse serum, remaining intact for 120 min at 37 °C, as determined by radio-TLC (Fig. S3 & Table S1 in the SI). This is consistent with similar observations of diethylene glycol-bearing esters [^18^F]Rho6G-DEG-F ([^18^F]1) and [^18^F]RhoB-DEG-F, and in contrast with 2-[^18^F]fluoroethyl RhoB (Fig. 1), which was previously found to be unstable in mouse serum.^25^

*Ex vivo* radio-metabolite analysis was conducted in two rats (one male and one female), with plasma samples processed with 8 M urea to denature proteins and release any bound tracer or metabolites prior to solid-phase extraction, followed by in-line radio-HPLC analysis. See HPLC D, Program F for a description of the column-switching apparatus used.^26^ Radio-HPLC traces of these experiments at 15 and 60 min post injection of tracer into both animals are shown in Fig. 4. In this method, any free [^18^F]fluoride and polar materials that do not trap on the plug of HLB sorbent elutes in the first four minutes, followed by an eight-minute gradient separation of remaining metabolites from intact tracer ([^18^F]6*t*_R_ = 11.5 min). Differences in the radiometabolic profiles of the two rats were observed (Table 2). In both sexes, a portion of the radioactivity is in the form of [^18^F]fluoride salts or highly polar metabolites, observed in the HPLC traces as two peaks between 0.85–2.75 min. In the female, intact [^18^F]6 decreased from 46% to 34% over 15–60 min, while in males the tracer decreased from 45% to 32% in the same time period. In addition, a radiometabolite of intermediate polarity is clearly visible at ∼7.3 min in the 15 min HPLC trace of the male (Fig. 4B). In contrast, the same metabolite was not observed in the female until 60 min post-injection.

**Fig. 4:**
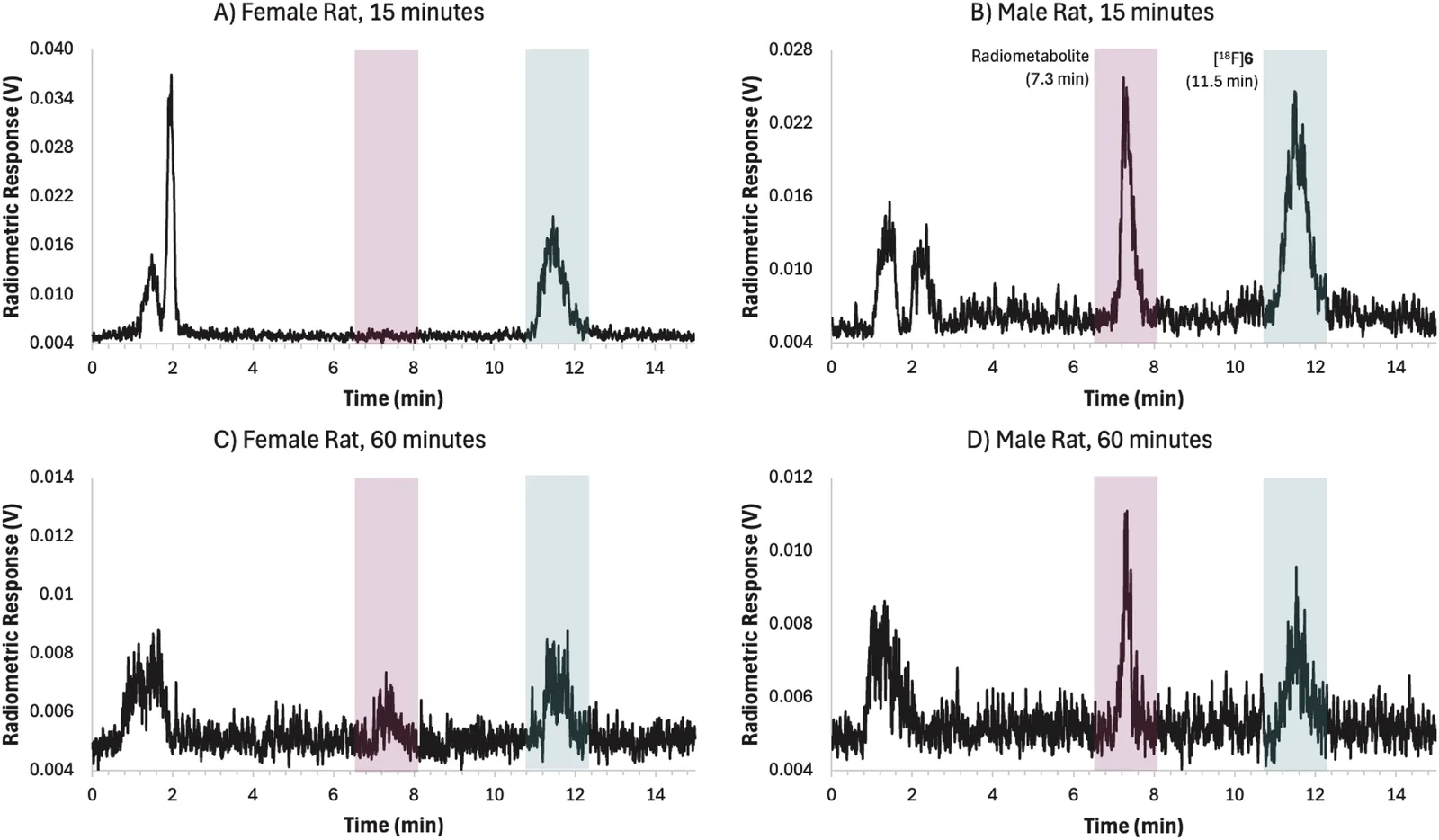
HPLC traces from *ex vivo* radiometabolite analysis at 15 minutes in a female (A) and male (B) rat, as well as 60 minutes in a female (C) and male (D) rat (HPLC D, Program F). The *t*_R_ of intact [^18^F]6 is ∼11.5 min (blue), and *t*_R_ of the radiometabolite is ∼7.3 min (pink).

**Table 2:** HPLC radiometabolite analysis of [^18^F]6 in male and female rats

Time	Male			Female		
	Activity eluted 0.85–2.75 min	Metabolite (∼7.3 min)	[^18^F]6 (∼11.5 min)	Activity eluted 0.85–2.75 min	Metabolite (∼7.3 min)	[^18^F]6 (∼11.5 min)
15 min	29%	26%	45%	54%	None observed	46%
30 min	—a	—a	—a	67%	None observed	33%
60 min	44%	24%	32%	47%	19%	34%

a Data not acquired due to HPLC failure.

### Lipophilicity of [^18^F]Rho6G-DEG-Py-F

The distribution coefficient (log *D*_7.4_) of [^18^F]6 between octanol and PBS (pH 7.4) was determined *via* a previously reported method for ^18^F-rhodamines.^6^ [^18^F]6 was found to have a lower log *D*_7.4_ (1.82) than the reported value of 1.96 for [^18^F]1,^6^ which reflects a small decrease in lipophilicity brought about by the presence of the 2-fluoropyridine group.

### Small animal PET imaging

Small animal PET imaging (µPET) was carried out on Sprague–Dawley rats (*n* = 8; 4 male, 4 female) injected with [^18^F]6. Representative images at later points (summed over 90–120 min) are shown in Fig. 5. These images demonstrate high and persistent myocardial uptake with good contrast between the myocardium, blood, liver, bones and lungs. Bone uptake localized in the head of the humerus and hips is consistent with uptake into red marrow^27^ rather than the more diffuse osteoblastic accumulation that is the hallmark of free ^18^F-fluoride. Time-activity curves (Fig. 6) and heart : liver (H : L) ratios reveal rapid liver washout with stable cardiac retention over 120 min, with standardized uptake values (SUVs) of ∼1 g mL^−1^ over the time course of the experiment. Promising H : L ratios were observed at late time points but exhibited some male/female sex bias. At 55 min, average H : L ratios were 1.7 and 1.2 for male and female rats respectively. At the 115 min timepoint, H : L ratio in male rats improved to 3.7 in males and 2.5 in females. Although care must be taken when comparing radiopharmaceuticals made at separate sites and using different radiopharmaceutical production methods, [^18^F]6 concentration in the myocardium over 60 min in this study was similar to earlier reported results with low molar activity [^18^F]1 obtained by the manual, two-step method (Table 3, row 1) [69 ± 5 kBq per cm^3^ (4 female rats)^6^*vs.* 68 ± 16 kBq per cm^3^ (average of 4 female & 3 male rats)]. However, the H : L ratios at 55 min reported for [^18^F]1 by both the direct and indirect manual approaches were higher than [^18^F]6 (Table 3), rows 1 & 2 *vs.* row 10, suggesting that [^18^F]1 clears more quickly from rat livers. Comparison of the two tracers at later timepoints (60–120 min) awaits additional µPET studies with [^18^F]1, but it does indicate that [^18^F]1 and perhaps other ^18^F-rhodamines might benefit from delayed PET acquisition protocols.

**Fig. 5:**
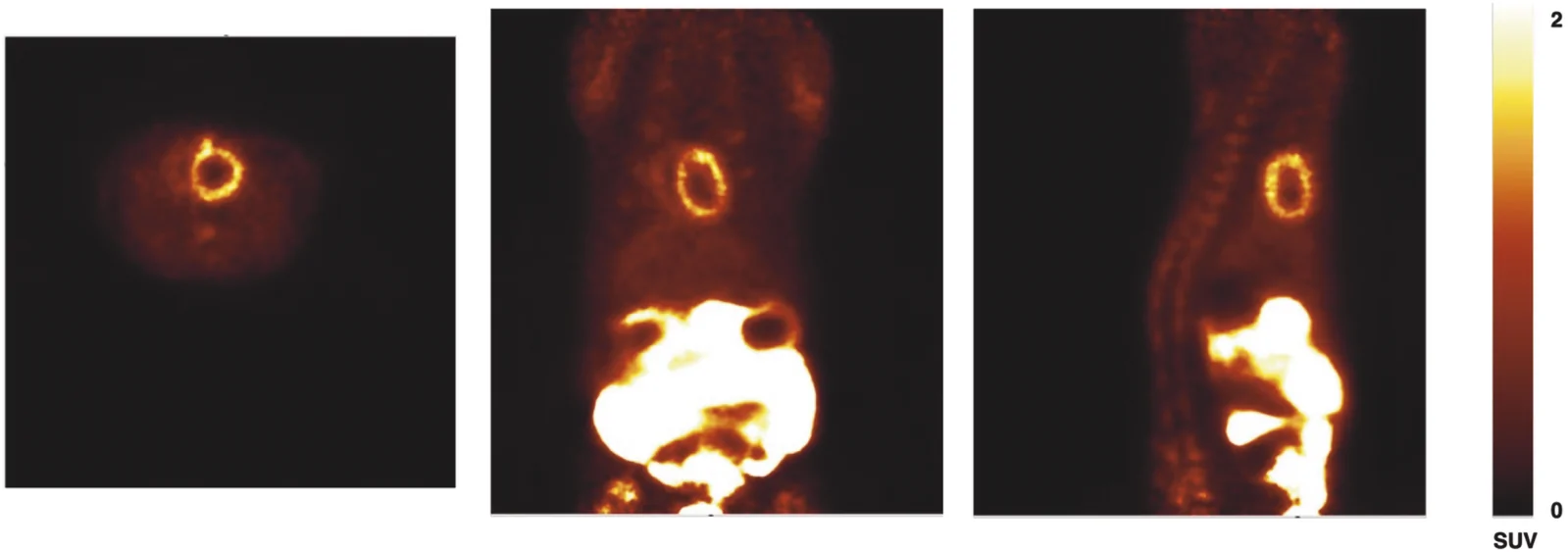
Micro-PET images of [^18^F]Rho6G-DEG-Py-F ([^18^F]6) in a female rat. Images are composite over 90–120 min. Left: transverse plane; Middle: coronal plane; Right: sagittal plane.

**Fig. 6:**
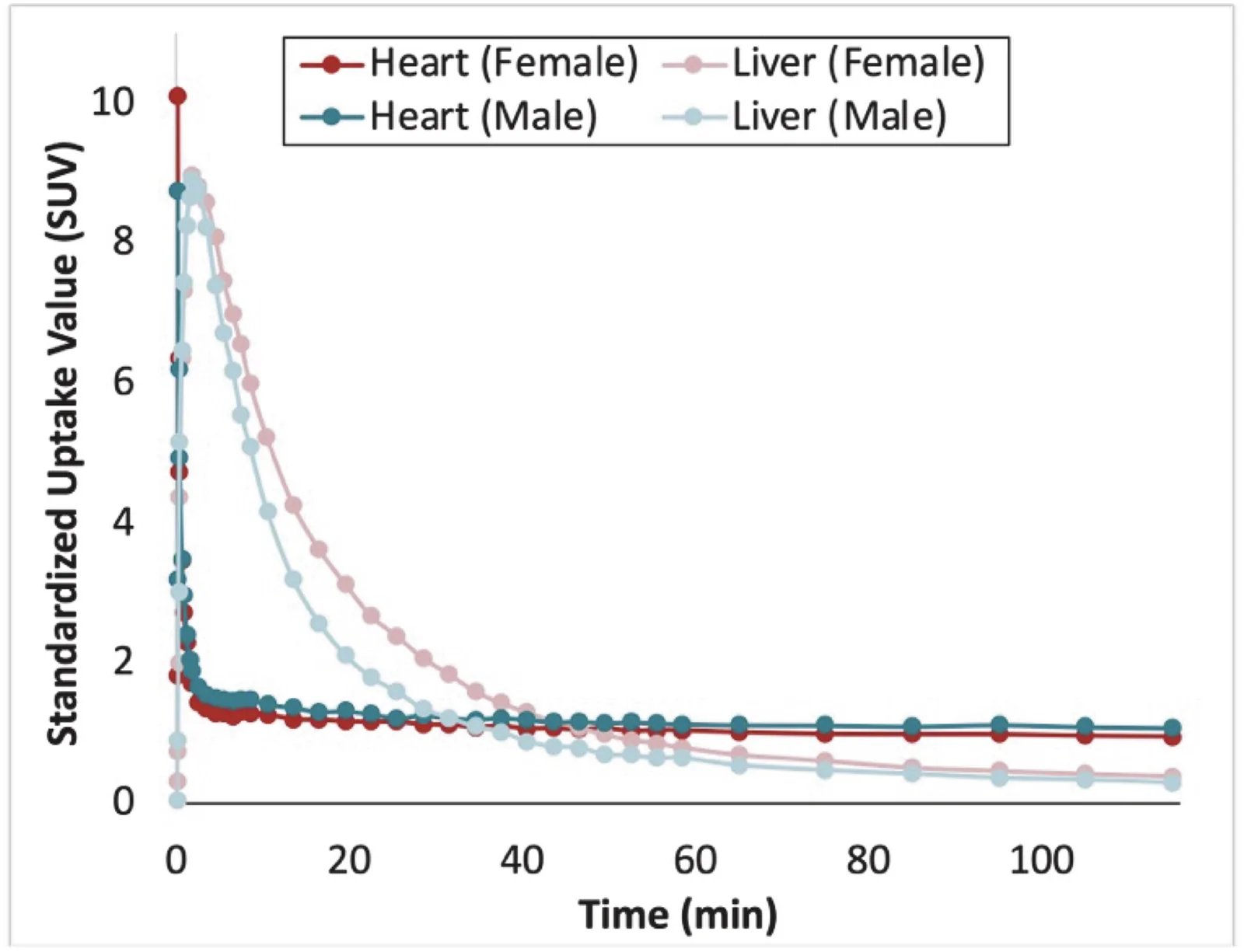
PET-derived time–activity curves of [^18^F]Rho6G-DEG-Py-F uptake into male and female rat hearts.

**Table 3:** Comparison between selected ^18^F-rhodamine radiosyntheses

Row	Rhodamine	Leaving group	Synthesis method	Synthesis type	%DC-RCY	*A*_m_ (GBq per µmol)	log *D*_7.4_	H : L @ 55 min
1 (ref. 6)	[^18^F]1	—	2-Step	Manual	19 ± 1, *n* = 3	0.32	1.96 ± 0.05, *n* = 4	4.5
2 (ref. 19 and 20)	[^18^F]1	OTs (2)	1-Step	Manual	9–21, *n* = 6	7–35, *n* = 6	—	2.9^a^
3 (ref. 19)	[^18^F]1	OTs (2)	1-Step	Automated	3–9, *n* = 3	122–256, *n* = 3	—	—
4 (ref. 11)	[^18^F]RhoB-DEG-F	—	2-Step	Manual	19 ± 1, *n* = 3	0.31	1.90 ± 0.01, *n* = 3	4.1
5 (ref. 25)	[^18^F]fluoroethylRhoB	—	2-Step	Manual	35	1.3	—	∼1.0^b^
6 (ref. 20)	[^18^F]Rho6G-pip-Ar-F	—	2-Step	Manual	8–21, *n* = 3	0.8–3.6, *n* = 3	1.56 ± 0.10, *n* = 4	0.2
7 (ref. 20)	[^18^F]RhoB-pip-Ar-F	—	2-Step	Manual	4–16, *n* = 3	0.6–4.9, *n* = 3	1.64 ± 0.06, *n* = 4	0.1
8^c^	[^18^F]6	NO_2_ (5)	1-Step	Manual	3–31, *n* = 9	1–50, *n* = 9	1.82 ± 0.01, *n* = 3	—
9^c^	[^18^F]6	NMe_3_^+^ (8)	1-Step	Manual	1–7, *n* = 6	23–41, *n* = 6	—	—
10^c^	[^18^F]6	NO_2_ (5)	1-Step	Automated	6–9, *n* = 3	39–88, *n* = 3	—	1.7/1.2^d^
11^c^	[^18^F]6	NMe_3_^+^ (8)	1-Step	Automated	0.7, *n* = 2	85–144, *n* = 2	—	—

a From ref. 20.

b Ratio estimated from a time–activity curve (kBq per cm^3^*vs.* time) of a single female rat.

c This work.

d Male/female respectively. At 115 min, H : L ratios improved to 3.7/2.5 respectively.

Following successful molecular imaging studies in rats, we evaluated [^18^F]6 in mice (*n* = 8; 4 male, 4 female). Comprehensive ^18^F-rhodamine radiopharmaceutical discovery efforts would be greatly facilitated by the use of mice in preclinical imaging, as most modern µPET scanners can image multiple mice at once through the use of four-bed “hotel”-type pallets. This represents the second time an ^18^F-rhodamine ester has been evaluated in this species using PET, despite some exhibiting stability in mouse serum (*i.e.* [^18^F]1). Previously, 2-[^18^F]fluoroethyl rhodamine B (Fig. 1) injections in mice yielded µPET scans showing significant gall bladder uptake, and subsequent *ex vivo* radio-TLC assay suggested that the major radioactive species in bile was [^18^F]fluoroethanol.^24^ Unfortunately, µPET images of [^18^F]6 in mice also showed little retention in myocardium (see Fig. S4 for representative PET images). In addition, [^18^F]6 was rapidly cleared from liver, suggesting that this tracer is hydrolyzed in mouse hepatocytes. SUV values ranged from 1.3 ± 0.5 to 0.3 ± 0.1 g mL^−1^ between 60 and 120 min, with H : L ratios of 0.1 ± 0.1 at 60 min, and 1.0 ± 0.3 at 120 min, in all mice. Differences in murine esterase expression and consequentially hydrolase activity could easily account for the observed differing clearance patterns between rats and mice.^28^ Furthermore, similar metabolic behaviour might be expected from [^18^F]1 and other candidate ^18^F-rhodamines possessing an ester bond. More unexpected was the radioactivity found substantially distributed to the skeletal structure of mice, suggesting radio-defluorination of the pyridine ring, a phenomenon more commonly associated with [^18^F]fluoroaliphatic PET tracers.^21,29^

## Experimental

### General information

A description of the chemicals, media and consumables used in the study, along with the HPLC systems and elution programs, can be found in the SI document.

### Non-radioactive synthesis

Synthesis of bifunctional diethylene glycol chains 9, 10, 11, as well as 2-dimethylamino-3-hydroxypyirdine (12) is described in the SI document.

#### Rhodamine 19 lactone (4)

Based on synthesis by Afonso *et al.*,^7^ rhodamine 6G chloride (3; 4.198 g; 8.76 mmol) was added to a 50 mL round bottom flask and heated to ∼250 °C while spinning in a Kugelrohr apparatus under vacuum. After 5 hours, the flask was cooled to room temperature to afford rhodamine 19 lactone (4; 3.436 g; 95%) as a pale purple powder. Silica gel TLC and ^1^H-NMR suggested clean conversion to 4 and the product was used without further purification. Silica gel TLC *R*_f_ (8 : 2 CH_2_Cl_2_–MeOH) = 0.8. ^1^H NMR (600 MHz, CDCl_3_) *δ* 1.32 (s, 6H), 1.93 (s, 6H), 3.23 (d, *J* = 7.2 Hz, 4H), 3.59 (s, 2H), 6.35 (s, 2H), 6.40 (s, 2H), 7.15 (d, *J* = 7.5 Hz, 1H), 7.60 (d, *J* = 27.0 Hz, 2H), 8.01 (d, *J* = 7.6 Hz, 1H). HR-MS calcd for C_26_H_26_N_2_O_3_: *m*/*z* 415.1940. Found: *m*/*z* 415.2059 [M]^+^.

#### Rho6G-DEG-Py-NO_2_ (5)

Rhodamine 19 lactone (4; 191 mg, 0.46 mmol), compound 9 (211 mg, 0.55 mmol, 1.2 eq.) and DIPEA (0.36 mL, 2.07 mmol, 4.5 eq.) were combined in a 4 mL amber V-vial and dissolved in MeCN (3 mL). The vial was sealed with a PTFE-lined cap and the reaction mixture was refluxed (90 °C) for 21 h, with stirring. The cap was punctured with two vent needles (18 gauge) and the temperature was increased to 130 °C over 10 min. The reaction mixture was held at this temperature for an additional 10 min, during which time the solvent and DIPEA evaporated. The vial was cooled to room temperature, and the crude product was purified *via* column chromatography with neutral alumina [9 : 1 ethyl acetate–MeOH] to afford 172 mg of 5 as a bright pink powder (50%). Silica gel TLC *R*_f_ (4 : 1 CH_2_Cl_2_–MeOH) = 0.47. ^1^H NMR (600 MHz, CDCl_3_) *δ* 1.41 (t, *J* = 7.2 Hz, 6H), 2.28 (d, *J* = 1.0 Hz, 6H), 3.48–3.51 (m, 2H), 3.56 (dt, *J* = 7.5, 5.8 Hz, 4H), 3.66 (dd, *J* = 5.2, 3.6 Hz, 2H), 4.12–4.15 (m, 2H), 4.16–4.21 (m, 2H), 6.68 (s, 2H), 6.77 (d, *J* = 1.2 Hz, 2H), 7.27 (m, 1H), 7.60 (dd, *J* = 8.4, 4.5 Hz, 1H), 7.70 (dd, *J* = 8.4, 1.3 Hz, 1H), 7.76 (ddd, *J* = 14.0, 7.6, 1.5 Hz, 2H), 8.09 (dd, *J* = 4.5, 1.3 Hz, 1H), 8.30 (dd, *J* = 7.7, 1.6 Hz, 1H). ^13^C NMR (151 MHz, CDCl_3_) *δ* 14.08, 18.57, 38.72, 60.54, 64.59, 69.13, 69.31, 69.87, 94.02, 113.77, 124.67, 126.22, 128.71, 129.26, 130.17, 130.36, 131.70, 133.01, 134.12, 139.62, 147.24, 156.06, 156.68, 157.27, 165.26. HR-MS calcd for C_35_H_37_N_4_O_7_^+^: *m*/*z* 625.2657. Found: *m*/*z* 625.2672 [M]^+^.

#### Rho6G-DEG-Py-NMe_2_ (7)

Rhodamine 19 lactone (4; 202 mg, 0.49 mmol), compound 10 (221 mg, 0.58 mmol, 1.2 eq.) and DIPEA (0.38 mL, 2.18 mmol, 4.5 eq.) were combined in a 4 mL amber V-vial with MeCN (3 mL). The vial was sealed with a PTFE-lined cap and the reaction mixture was heated to reflux (90 °C) for 20 h, with stirring. The cap was punctured with two vent needles, then the temperature was increased to 130 °C over 12 minutes and held at this temperature for an additional 10 min. The vial was then cooled to room temperature, and the crude product was purified twice *via* column chromatography with neutral aluminium oxide (8 : 2 ethyl acetate–MeOH, then 9 : 1 ethyl acetate–MeOH) to afford 169 mg of 7 as a bright red solid (43%). Silica gel TLC *R*_f_ (4 : 1 CH_2_Cl_2_–MeOH) = 0.60. ^1^H NMR (600 MHz, MeOD) *δ* 1.34 (t, *J* = 7.2 Hz, 6H), 2.10 (d, *J* = 1.2 Hz, 6H), 2.86 (s, 6H), 3.37–3.40 (m, 2H), 3.48 (qd, *J* = 7.2, 2.2 Hz, 4H), 3.60–3.63 (m, 2H), 3.96–4.01 (m, 2H), 4.08–4.11 (m, 2H), 6.78 (dd, *J* = 7.8, 5.0 Hz, 1H), 6.88 (d, *J* = 1.2 Hz, 2H), 6.91 (s, 2H), 7.15 (dd, *J* = 7.8, 1.5 Hz, 1H), 7.40 (dd, *J* = 7.5, 1.3 Hz, 1H), 7.73 (dd, *J* = 5.0, 1.5 Hz, 1H), 7.80 (td, *J* = 7.7, 1.3 Hz, 1H), 7.87 (td, *J* = 7.6, 1.4 Hz, 1H), 8.32 (dd, *J* = 7.9, 1.4 Hz, 1H). ^13^C NMR (151 MHz, DMSO) *δ* 13.62, 17.46, 37.96, 40.06, 40.31, 64.44, 67.19, 68.03, 68.75, 93.54, 112.93, 115.27, 118.89, 125.36, 128.46, 129.33, 130.32, 130.42, 130.96, 133.30, 133.46, 138.41, 144.82, 152.27, 155.75, 156.77, 157.01, 164.78. HR-MS calcd for C_37_H_44_N_4_O_5_^+^: *m*/*z* 623.3228. Found: 623.3248 *m*/*z* [M]^+^.

#### Rho6G-DEG-Py-NMe_3_^+−^OTf (8)

Rho6G-DEG-Py-NMe_2_ (7; 80 mg, 0.12 mmol) was added to a dry 15 mL conical polypropylene centrifuge tube (Falcon®) charged with Ar, and dissolved in anhydrous CH_2_Cl_2_ (1.5 mL). Methyl triflate (0.133 mL, 1.20 mmol, 10 eq.) was added over the course of the reaction. The reaction proceeded at room temperature with stirring for 25 h, after which the tube was centrifuged (2500×*g*; 3 min), and the supernatant was removed. The precipitate was washed with ice-cooled anhydrous CH_2_Cl_2_ (4 × 4 mL) to yield 80.1 mg of 8 (70%) as a red-purple solid. ^1^H NMR (600 MHz, MeOD) *δ* 1.37 (s, 6H), 2.10 (s, 6H), 3.49–3.55 (m, 6H), 3.60 (s, 9H), 3.77–3.81 (m, 2H), 4.14–4.19 (m, 2H), 4.32–4.37 (m, 2H), 6.86 (d, *J* = 1.3 Hz, 2H), 6.95 (s, 2H), 7.41 (dd, *J* = 7.5, 1.3 Hz, 1H), 7.67 (dd, *J* = 8.3, 4.5 Hz, 1H), 7.85 (ddtd, *J* = 23.5, 15.4, 7.6, 1.4 Hz, 3H), 8.16 (dd, *J* = 4.5, 1.3 Hz, 1H), 8.32 (dd, *J* = 8.0, 1.3 Hz, 1H). ^13^C NMR (151 MHz, MeOD) *δ* 14.05, 17.52, 39.46, 49.57, 54.68, 65.44, 69.77, 69.86, 70.29, 94.82, 114.87, 126.38, 126.82, 129.74, 130.07, 131.23, 131.48, 131.70, 132.31, 134.31, 135.33, 140.27, 148.65, 157.68, 159.00, 159.54, 166.47. ^19^F NMR (565 MHz, MeOD) *δ* −80.11. HR-MS calcd for C_38_H_45_N_4_O_5_: 319.1729 *m*/*z*. Found: 319.1735 *m*/*z* [M]^+^/2.

#### Rho6G-DEG-Py-F (6)

Rhodamine 19 lactone (4; 0.200 g, 0.48 mmol), 11 (0.206 g, 0.57 mmol) and DIPEA (0.376 mL, 2.16 mmol) were combined in a 4 mL amber V-vial with MeCN (3 mL). The vial was closed tightly with a PTFE-lined cap and the reaction mixture was refluxed at 90 °C for 20 h, with stirring. The vial was then punctured with two vent needles, the temperature was increased to 130 °C over 10 min and held at 130 °C for an additional 10 min, until remaining solvent and DIPEA were distilled off. The vial was then cooled to room temperature, producing a dark purple oil. The crude product was vacuum-filtered through a short column of silica gel with 3 : 2 toluene–EtOH. Two fractions were collected and concentrated *in vacuo* to afford a pink-purple solid in 93% and 95% purity by HPLC (HPLC A, Program E). Total collected material was 0.271 g (73%, factoring for purity). A fraction of this material was further purified to 97% by column chromatography with neutral aluminium oxide [9 : 1 ethyl acetate–MeOH] for use building a standard curve to estimate [^18^F]6 molar activity. Silica gel TLC *R*_f_ (3 : 2 toluene–EtOH) = 0.27; *R*_f_ (4 : 1 CH_2_Cl_2_–MeOH) = 0.68. ^1^H NMR (600 MHz, CDCl_3_): *δ* 1.41 (t, *J* = 7.2 Hz, 6H), 2.26 (s, 6H), 3.52–3.57 (m, 6H), 3.69–3.74 (m, 2H), 4.10–4.13 (m, 2H), 4.15–4.18 (m, 2H), 6.66 (s, 2H), 6.75 (s, 2H), 7.13 (dd, *J* = 7.8, 4.9 Hz, 1H), 7.27–7.29 (m, 1H), 7.33 (ddd, *J* = 9.9, 7.9, 1.6 Hz, 1H), 7.72–7.80 (m, 3H), 8.32 (dd, *J* = 8.0, 1.4 Hz, 1H). ^13^C NMR (600 MHz, CDCl_3_): *δ* 14.00, 18.46, 38.69, 64.52, 69.02, 69.16, 69.41, 93.91, 113.65, 122.09, 123.47, 126.26, 128.60, 130.01, 130.22, 131.66, 133.00, 134.30, 137.74, 142.12, 142.29, 153.09, 154.67, 156.10, 156.64, 157.22, 165.15. ^19^F NMR (471 MHz, CDCl_3_) *δ* −83.96. HR-MS calcd for C_35_H_37_FN_3_O_5_^+^: 598.2712 *m*/*z*. Found: 598.2728 *m*/*z* [M]^+^.

### Radiochemistry

#### Preparation of [^18^F]fluoride

For manual radiochemistry, 740–1850 MBq (20–50 mCi) of no-carrier-added [^18^F]fluoride was produced on-site by proton bombardment of [^18^O]water [^18^O(p,n)^18^F reaction] on a GE 16.5 MeV PETtrace negative ion cyclotron. For automated radiosynthesis, 18.5–37 GBq (0.5–1 Ci) of no-carrier added [^18^F]F^−^ was produced on a Scanditronix MC-17 cyclotron and transferred in [^18^O]H_2_O to an automated radio-synthesizer (GE TRACERlab FX2 N) in a lead-shielded hot cell.

### Radiosynthesis of [^18^F]FRho6G-DEG-Py ([^18^F]6)

#### Representative manual radiosynthesis

This procedure corresponds to Run 9 in Table 1. [^18^F]F^−^ was extracted from [^18^O]H_2_O *via* immobilization on a small QMA anion-exchange cartridge (12 mg; bicarbonate form; General Science Inventions, Saskatoon, CA), which was previously activated with water (10 mL). The activity was eluted from the sorbent into a glass conical vial (3 mL) with a solution of TEAHCO_3_ (1.38 mg) in 7 : 3 MeCN : H_2_O (1 mL). Solvent was removed by azeotropic distillation at 110 °C under an argon stream. An additional portion of dry MeCN (1 mL) was added to the residue, and another evaporation step was carried out at 110 °C. A third portion of MeCN was added (1 mL) and again concentrated at 110 °C (25 min total ‘drydown’ time). The precursor (5; 0.5 mg) in anhydrous DMSO (0.5 mL) was added. The vial was sealed and heated at 160 °C for 20 min. After cooling, an aliquot was removed for radio-TLC (silicic acid, 20% MeOH in CH_2_Cl_2_, 70% RCC), then the bulk mixture was quenched with an aqueous solution of 0.1% TFA (1 mL). After semi-preparative HPLC purification (HPLC A, Program B, *t*_R,RAD_ = 28.95 min, *t*_R,530nm_ = 29.86 min), the collected product was diluted with water (50 mL) and trapped on a Sep-Pak Light tC18 cartridge [previously activated with EtOH (5 mL) and then washed with water (10 mL)]. [^18^F]6 was eluted with EtOH (2 mL), and the solvent removed at 110 °C under Ar. The final product was formulated in 10% EtOH and 0.9% saline and assayed by analytical HPLC (HPLC A, Program C, *t*_R,RAD_ = 10.25 min) to determine chemical and radiochemical purity. The integrated UV signal (530 nm) in this trace observed co-eluting with radioactivity was compared with a mass standard curve for the estimation of molar activity (50 GBq per µmol at EOS). Co-injection of a small sample of the final formulation with non-radioactive standard 6 under the same HPLC conditions was used to verify product identity (Fig. S5). [^18^F]6 was obtained in a final activity yield of 81.4 MBq (2.2 mCi); preparative radiochemical yield was 5% (14% DC) from SOS. Total synthesis time was 162 min from SOS.

#### Representative automated radiosynthesis

[^18^F]F^−^ in [^18^O]H_2_O was transferred to a GE Tracerlab FX2 N automated synthesis unit where [^18^F]F^−^ was extracted from [^18^O]H_2_O *via* immobilization on a QMA anion-exchange cartridge (12 mg; bicarbonate form), which was previously activated with water (10 mL). The activity was eluted into a reaction vessel with a solution of TEAHCO_3_ (1.38 mg) in 7 : 3 MeCN : H_2_O (1 mL). The reaction vessel was heated to 85 °C under vacuum while stirring, followed by adding 1 mL MeCN and heating at 100 °C to evaporate the remaining extraction solvent. The reactor was then cooled to 70 °C before adding precursor 5 (0.5 mg in 0.5 mL anhydrous DMSO), and the reaction mixture was heated at 160 °C, with stirring. After 20 min, the reactor was cooled to 50 °C and the crude product mixture was quenched with 0.1% TFA in water (4 mL) before being loaded onto semi-preparative HPLC (HPLC B, Program B, *t*_R,RAD_ = 21.0 min). The collected eluate was diluted with water (50 mL) and trapped on a Sep-Pak Light tC18 cartridge, which was previously activated with EtOH (5 mL), then washed with water (10 mL). The cartridge was washed with water (10 mL) and [^18^F]6 was eluted with EtOH (2 mL) followed by 0.9% saline (10 mL), which was filtered (0.22 µm) into a final vial. Chemical and radiochemical purity of [^18^F]6 was verified by analytical HPLC (HPLC C, Program D, *t*_R,RAD_ = 11.38), and the integrated UV signal at 254 nm in this trace observed co-eluting with radioactivity was compared with a mass standard curve for the estimation of molar activity (50 GBq per µmol at EOS). Product identity was confirmed *via* co-injection with non-radioactive standard 6. Fifty-one mCi (1887 MBq) of product was obtained, resulting in a preparative radiochemical yield of 6% (9% DC) from SOS. Total synthesis time was 78 min from SOS.

### Serum stability studies

Three 20 µL aliquots of [^18^F]6 in 10% ethanol/PBS (40–100 µCi) were added to PBS, rat serum or mouse serum (200 µL each) in a microcentrifuge tube previously equilibrated to 37 °C. The tubes were briefly vortexed, and the samples were placed back in the heating block at 37 °C. At selected time points (15, 60 and 120 min) enzymatic hydrolysis was quenched with the addition of cold (4 °C) absolute ethanol (1 mL). The samples were cooled in ice to precipitate serum proteins, centrifuged (15 min, 2500×*g*, 4 °C), and the supernatant was analyzed by radio-TLC (silica gel, 4 : 1 CH_2_Cl_2_ : MeOH). The percentage of intact [^18^F]6*vs.* total radioactivity at each time point was calculated from the radio-chromatograms.

### Partition coefficient (log *D*_7.4_)

[^18^F]Rho6G-DEG-Py-F (105–125 µCi) in 20 µL 10% EtOH/PBS was added to a mixture of PBS (pH 7.4; 480 µL) and octanol (500 µL) in microcentrifuge tubes. Samples were vortexed for 1 min, then centrifuged for 5 min (2500×*g*). From each sample, three 100 µL aliquots of both the PBS and the organic layer were transferred into separate microcentrifuge tubes, and the radioactivity in each tube was measured with a gamma counter.

### Animal studies

#### PET acquisition in rats and mice

Rodent experiments were performed in accordance with animal protocols approved by the Animal Care and Use Committee at the Centre for Addiction and Mental Health (CAMH AUP#891). Adult Sprague–Dawley rats [3–5 months old, *n* = 8, 4 male (M)/4 female (F)] and C57BL6 mice (10 months old, *n* = 8, 4 males/4 females) from Charles River Laboratories were used for µPET imaging with [^18^F]Rho6G-DEG-Py-F. PET images were acquired using a nanoScan™ PET/CT scanner (Mediso, Budapest, Hungary). The animals were anesthetized by isoflurane (4–5% induction; O_2_ rate: 2 L min^−1^) and catheterized in the lateral tail vein. Following insertion, the catheter was flushed with heparinized saline (30 IU per mL). The animal was transferred to the scanner bed in prone position, the head immobilized in a flat skull position for the duration of the acquisition using built-in bite bars. The scanner bed temperature was initially set at 40 °C, but was subject to alteration based on an animal's body temperature during the experiment. Anaesthesia was maintained throughout the scanning procedure (isoflurane: 1.5–2%; O_2_ rate: 1–2 L min^−1^) and the animal's body temperature and respiration parameters were closely monitored. For mice, a quad-mice pallet was used with the male and female animals arranged alternatively for the two scans. A scout CT was first acquired for PET field-of-view (FOV) positioning, then a material map CT image was acquired for PET corrections of attenuation and scatter, and for PET/CT co-registration to define anatomic regions of interest. Dynamic emission list mode scans of 120 min with an energy window of 400–600 keV were started immediately following bolus injection of the radiotracer through the lateral tail vein (7–28 MBq for rats and 3–8 MBq for mice).

#### PET image analysis

Acquired list mode data sets were sorted into 39 three-dimensional (3D) (3 × 5 s, 3 × 15 s, 3 × 20 s, 7 × 60 s, 17 × 180 s, and 6 × 600 s) true sinograms (ring difference 84). The 3D sinograms were converted in 2D sinograms using Fourier rebinning with corrections for detector geometry, efficiencies, attenuation, and scatter before image reconstruction using 2D filtered back-projection with a Hann filter at a cut-off of 0.50 cm^−1^. All data were corrected for dead time and were decay-corrected to the start of acquisition. Iteratively reconstructed images with the manufacturer's proprietary 3D algorithm (6 subsets, 4 iterations) were used for PET and CT co-registration, for organ volume-of-interest (VOI) definition, and for image presentation. Filtered back-projection images were used to extract organ time–activity curves (TACs) with PMOD software (version 4.203, Zurich, Switzerland). Standardized Uptake Values (SUVs) were calculated by normalizing regional radioactivity for injected radioactivity and body weight.

#### Plasma metabolites analysis

For arterial blood sampling in rats, a catheter was placed in the ventral tail artery and at 15, 30 and 60 min post-injection of [^18^F]Rho6G-DEG-Py-F through the lateral tail vein catheter, and blood samples (0.3 mL) were collected into an EDTA-coated microvette (Sarstedt). Whole blood (10 µL) was drawn into a pre-weighed tube and the remaining blood was centrifuged at 2500×*g* for 5 min to collect plasma. Radioactivity in 10 µL of plasma, together with the whole blood samples, were measured by gamma counting to calculate activity concentrations (kBq per mL), using density values of 1.0262 and 1.056 g mL^−1^ for plasma and whole blood, respectively, and to calculate the plasma-to-blood ratio. The rest of the plasma was diluted with isotonic saline to 4.5 mL and mixed with solid urea (2.12 g) to a concentration of 8 M urea. This mixture was directly injected onto an automated, custom-built, integrated SPE/HPLC column-switching system^26^ (See HPLC D, Program F in the SI for details). Area-under-the-curve of each radiometric peak was computed to determine the percent parent radiotracer in the plasma (*i.e.*, parent fraction).

## Conclusions

A novel ^18^F-labelled rhodamine was synthesized and evaluated in rats and mice as a candidate myocardial perfusion PET imaging agent. 2-[^18^F]fluoropyridine-bearing rhodamine 6G analogue [^18^F]Rho6G-DEG-Py-F ([^18^F]6) demonstrates comparable preclinical utility to [^18^F]Rho6G-DEG-F ([^18^F]1), a first-generation [^18^F]fluoroaliphatic radiotracer, while also overcoming key limitations associated with the synthesis of its radiolabelling precursors. Using manual techniques, [^18^F]6 was reliably synthesized from both 2-nitropyridine-bearing precursor 5 (best DC-RCY = 31%, *A*_m_ = 17 ± 19 GBq per µmol) and 2-trimethylammoniumpyridine-bearing precursor 8 (best DC-RCY = 7%, *A*_m_ = 33 ± 8 GBq per µmol) *via* nucleophilic aromatic ^18^F-fluorination reactions. Automated production of [^18^F]6 using a TRACERLab FX2 N synthesizer yielded improvements to activity yield, radiochemical yield and molar activity. Thus, up to 1887 MBq (51 mCi) of tracer was obtained in a single production run, starting from 33.3 GBq (900 mCi) [^18^F]F^−^ and precursor 5. Best DC-RCY obtained was 9% (using 5), and best *A*_m_ was 144 GBq per µmol (using 8).

[^18^F]6 displays excellent stability in rat and mouse serum over 120 min. As previously observed for [^18^F]1, [^18^F]6 exhibits rapid myocardial uptake in µPET scans of healthy rats, with persistent retention (SUV_mean_ > 1.0 at 120 min) and high heart : liver ratios (3.7 male, 2.5 female at 120 min). Arterial blood sampling during µPET scans suggested differing radiometabolite profiles between a male and a female rat, although the percent of intact tracer at 60 min was very similar (32–34%). Although the differences observed between the two animals were distinct, firm conclusions regarding possible sex-based variation in [^18^F]1 metabolism will require further animal testing. In contrast with observations in rats, µPET images in mice revealed rapid metabolism and poor myocardial uptake. These observations highlight both sex- and species- dependent differences in heart uptake that suggest additional preclinical experimentation of both [^18^F]1 (in male animals) and [^18^F]6 (in larger animals) would be prudent.

## Author contributions

Maya Mahmood: methodology, formal analysis, investigation, writing – original draft, writing – review & editing, visualization, project administration. Emily Murrell: methodology, investigation, resources, writing – original draft, writing – review & editing, supervision. Seniha Ozyuncu: investigation. Junchao Tong: methodology, formal analysis, investigation, writing – original draft. Angelina Lui: investigation. James A. H. Inkster: conceptualization, methodology, resources, writing – original draft, writing – review & editing, visualization, supervision, project administration, funding acquisition.

## Conflicts of interest

There are no conflicts to declare.

## Supplementary Material

RA-OLF-D6RA08884H-s001

## Data Availability

Data supporting this article has been included as part of an electronic supporting information (SI) document, including HPLC methods and other instrumentation, synthetic details for compounds 9–12, HPLC traces related to radiosynthetic method development, [^18^F]6 serum and plasma stability data, spectroscopic data of non-radioactive compounds, and additional PET images in mice. Supplementary information is available. See DOI: https://doi.org/10.1039/d6ra08884h.
