# Supplementary material for: Synthesis and preclinical evaluation of a novel 2-[18F]fluoropyridine-bearing rhodamine for myocardial perfusion imaging with PET
Source: RSC Adv. 2026 Nov 5. Online ahead of print. doi: 10.1039/d6ra08884h (PMC13738243; doi:10.1039/d6ra08884h)
Supplement: Supplementary file 1 [file RA-OLF-D6RA08884H-s001.pdf]

Supplementary Information (SI) for RSC Advances.
This journal is © The Royal Society of Chemistry

SUPPORTING INFORMATION

Synthesis and preclinical evaluation of a novel 2-[^{18}F]fluoropyridine-bearing rhodamine for myocardial perfusion imaging with PET

~~Initial Evaluation of a Novel 2-[^{18}F]Fluoropyridine-Bearing Rhodamine for Myocardial Perfusion Imaging with PET~~

Maya Mahmood,[a] Emily Murrell,[b,c] Seniha Ozyuncu,[a] Junchao Tong,[b] Angelina Lui,[a] and James A.H. Inkster*[a]

[a] McMaster University, Department of Chemistry and Chemical Biology, Hamilton, ON, L8S 4M1, Canada
[b] Brain Health Imaging Centre, Centre for Addiction and Mental Health, Toronto, ON, M5T 1R8, Canada
[c] Department of Psychiatry, University of Toronto, Toronto, ON, M5T1R8, Canada

Table of Contents

Chemicals and media......3
General Instrumentation......3
HPLC systems and elution programs......3
Non-radioactive syntheses of compounds 9-12......6
2-(2-(2-Nitropyridin-3-yloxy)ethoxy)ethyl tosylate (9)......6
2-(2-(2-Dimethylaminopyridin-3-yl)oxy)ethyl tosylate (10)......6
2-(2-((2-Fluoropyridin-3-yl)oxy)ethoxy)ethyl tosylate (11)......7
2-Dimethylamino-3-hydroxypyridine (12)......7
Additional Figures (S1 - S5) and Table (S1)......9
Figure S1......9
Figure S2......10
Figure S3......11
Table S1......11
Figure S4......13
Figure S5......13
NMR and ESI Mass Spectra of Non-radioactive Compounds......14
Rhodamine 6G lactone (4)......14
2-(2-((2-Fluoropyridin-3-yl)oxy)ethoxy)ethyl tosylate (11)......15
Rho6G-DEG-Py-F (6)......17
2-(2-(2-nitropyridin-3-yloxy)ethoxy)ethyl tosylate (9)......19

Rho6G-DEG-Py-NO_2 (5) 20

2-dimethylamino-3-hydroxypyridine (12) 22

2-(2-((2-dimethylaminopyridin-3-yl)oxy)ethoxy)ethyl tosylate (10) 23

Rho6G-DEG-Py-NMe_2 (7) 24

Rho6G-DEG-Py-NMe_3^+ (8) 27

Chemicals and media

All solvents and reagents were purchased from Millipore Sigma, Fisher Scientific, or ChemScene, and used without further purification. Silica gel and alumina sorbents for flash chromatography were obtained from Zeochem Silica Materials (Quebec City, QC, Canada) and Millipore Sigma respectively. Quaternary methylammonium (QMA) anion-exchange cartridges (12 mg, bicarbonate form) for $[^{18}F]F^-$ trap-and-release were obtained from General Science Inventions, Inc. (Saskatoon, SK, Canada). Solid phase extraction (SPE) cartridges [Sep-Pak tC18 and C18 Light (both 145 mg), and Oasis HLB sorbent (30 *µ*m)] were obtained from Waters (Milford, MA, USA). Silicic Acid (SA)–impregnated glass microfiber thin-layer chromatography (iTLC) sheets were purchased from Molpure (Laguna Hills, CA, USA).

General Instrumentation

An Eckert & Ziegler AR-2000 TLC scanner was used to assay TLC plates.

Nuclear Magnetic Resonance (NMR) was performed on a Bruker AV 600 MHz spectrometer with autosampler, and chemical shifts (δ) were reported in ppm relative to deuterated solvent residual peaks.

Measurement of low-activity samples was carried out on a Perkin Elmer Wizard2 2470 gamma counter.

HPLC systems and elution programs

HPLC A. For manual experiments. Pump: Agilent 1100 Binary Pump. UV detection: 1200 series module diode array detector (DAD). Radiation detection: Carroll and Ramsey model 105S-1 single channel high-sensitivity radiation detector. Signal integrated *via* an Agilent 35900E dual channel interface module.

HPLC B. For automated experiments. Pump: Sykam GmbH S1122 HPLC pump. UV detection: Knauer AZURA® UVD 2.1S module.

HPLC C. Analytical. Pump: Advion AVANT Quat Gradient Pump. UV detection: Advion UV/Vis-DAD module. Radiation detection: Bioscan (Eckert & Ziegler, Germany).

HPLC D. For plasma metabolite experiments. Custom-built, automated HPLC integrated with a self-packed VICI Valco pre-column cartridge.[1] Pump: Advion AVANT Quat Gradient Pump. UV detection: Advion

UV/Vis-DAD module. Radiation detection: Bioscan (Eckert & Ziegler, Germany). Signals integrated *via* Advion Data Express software.

PROGRAM A. Column: Kinetex® 5 μm, F5 100Å, 250 x 10 mm. Solvent system: isocratic elution, 50:50 MeCN:H_2O containing 0.1% trifluoroacetic acid (TFA), flow rate = 3.5 mL/min. UV: 254, 530 nm.

PROGRAM B. Column: Kinetex® 5 μm, F5 100Å, 250 x 10 mm. Solvent system: isocratic elution, 45:55 MeCN:H_2O containing 0.1% TFA, flow rate = 3.5 mL/min. UV: 254, 530 nm.

PROGRAM C. Column: Kinetex® 5 μm, F5 100Å, 250 x 10 mm. Solvent system: gradient elution, 30% MeCN in H_2O containing 0.1% TFA for 1 min, raised to 100% over 10 min, hold for 3 min, flow rate = 3.5 mL/min. UV: 254, 530 nm.

PROGRAM D. Column: Luna® 5 μm, PFP(2) 100Å, 250 x 10 mm. Solvent system: gradient elution, 30% MeCN in H_2O containing 0.1% TFA for 1 min, raised to 100% over 10 min, hold for 3 min, flow rate = 1 mL/min. UV: 254, 530 nm.

PROGRAM E. Column: Kinetex® 5 μm, F5 100Å, 150 x 4.6 mm. Solvent system: gradient elution, 10% MeCN in H_2O containing 0.1% formic acid for 2 min, raised to 100% MeCN containing 0.1% formic acid over 15 min, hold for 3 min, flow rate = 1 mL/min. UV: 254, 530 nm.

PROGRAM F. Columns: An in-line, self-packed Oasis HLB SPE capture column, then a Gemini NX-C18, 5 μm, 250 x 4.6 mm HPLC column. Plasma radiometabolites were first trapped on the SPE cartridge using an eluent of 1% MeCN in 0.1 M aqueous ammonium formate [HCOONH₄] over 4 min to remove plasma proteins, then flow was reversed and directed onto the analytical HPLC column. A dynamic gradient was used: 40 to 95% MeCN in 0.1 M $HCOONH_{4(aq)}$ over 8 min, followed by wash of 1 min with 95% MeCN in 0.1 M $HCOONH_{4(aq)}$, and re-equilibration for 2 min with 1% MeCN in 0.1 M $HCOONH_{4(aq)}$. Total run time = 15 min. Flow rate = 1.8 mL/min. UV: 200–400 nm.

PROGRAM G. Column: Kinetex® 5 μm, F5 100Å, 150 x 4.6 mm. Solvent system: gradient elution, 10% MeCN in H_2O containing 0.1% formic acid for 2 min, raised to 70% over 18 min, then raised to 95% over 5 min, flow rate = 1 mL/min. UV: 254, 530 nm.

PROGRAM H. Column: Kinetex® 5 μm, F5 100Å, 150 x 4.6 mm. Solvent system: isocratic elution, 45:55 MeCN:H_2O containing 0.1% formic acid, flow rate = 1 mL/min. UV: 254, 530 nm.

PROGRAM I. Column: Kinetex® 5 μm, F5 100Å, 150 x 4.6 mm. Solvent system: isocratic elution, 45:55 MeCN:H_2O containing 0.1% TFA, flow rate = 1 mL/min. UV: 254, 530 nm.

Non-radioactive syntheses of compounds 9-12

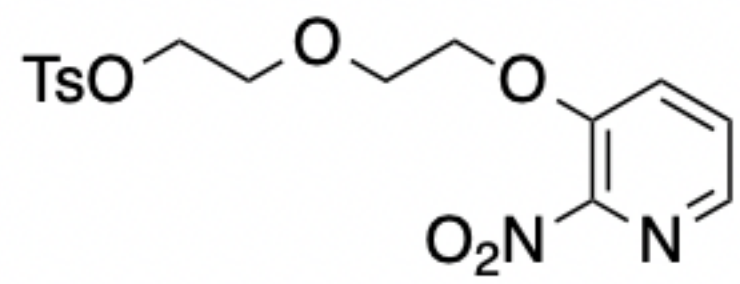

2-(2-(2-Nitropyridin-3-yloxy)ethoxy)ethyl tosylate (9). 2-Nitro-3-hydroxypyridine (500 mg, 3.56 mmol), diethylene glycol di(p-toluenesulfonate) (1.48 g, 3.57 mmol) and powdered K_2CO_3 (986.78 mg, 7.14 mmol, 2 eq) were added to a dry round bottom flask. Dry MeCN (60 mL) was added, and the reaction was refluxed under Ar for 6 hours. The Ar line was removed, and the reaction proceeded for 15 hours. After the reaction mixture was cooled to room temperature, saturated $NH_4Cl_{(aq)}$ (40 mL) was added. The solution was transferred to a separatory funnel and the product was extracted three times into ethyl acetate (3 x 40 mL). The combined organic portions were washed with brine (30 mL) and dried for 3 hours over magnesium sulfate. The product was filtered and concentrated *in vacuo*. Purification was conducted by silica gel column chromatography eluted with 1:2 hexanes:ethyl acetate to yield a clear oil (0.7 g, 51%). ^{1}H NMR (600 MHz, $CDCl_3$) δ 2.43 (s, 3H), 3.72~~7~~–3.77~~2~~ (m, 2H), 3.80~~6~~–3.86~~0~~ (m, 2H), 4.13~~7~~–4.17~~3~~ (m, 2H), 4.23~~7~~–4.27~~3~~ (m, 2H), 7.33 (d, J = 8.1 Hz, 2H), 7.53 (dd, J = 8.4, 4.5 Hz, 1H), 7.60 (dd, J = 8.4, 1.1 Hz, 1H), 7.77 (d, J = 8.3 Hz, 2H), 8.10 (dd, J = 4.5, 1.1 Hz, 1H). ^{13}C NMR (151 MHz, $CDCl_3$) δ 21.78, 69.32, 69.37, 69.62, 70.06, 124.75, 128.05, 128.80, 130.03, 133.03, 139.80, 145.09, 147.35, 149.25. LC LR-MS calcd for $C_{16}H_{18}N_2O_7S^+$: 383.08. Found $[M]^+$ 383.09.

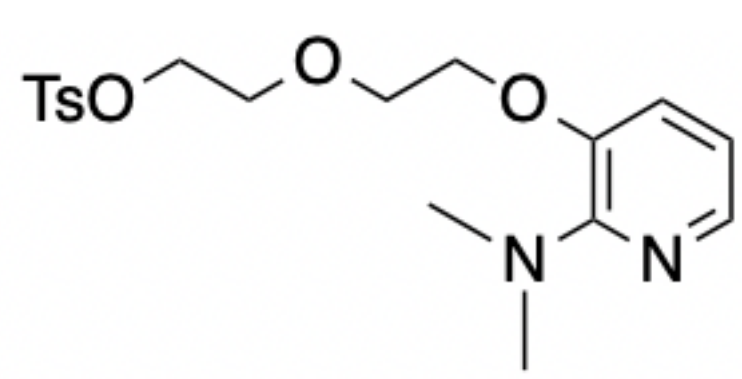

2-(2-(2-Dimethylaminopyridin-3-yl)oxy)ethyl tosylate (10). 2-dimethylaminopyridin-3-ol (**12**; 155 mg, 1.16 mmol) and diethylene glycol di(p-toluenesulfonate) (960 mg, 2.32 mmol, 2 eq) were added to a flame-dried 250 mL round bottom flask under Ar. The compounds were dissolved in dry MeCN (20 mL) and powdered K_2CO_3 (160 mg, 1.16 mmol) was added. The mixture was refluxed under Ar for 5 h, then cooled to room temperature and quenched with ethyl acetate (20 mL). The reaction was then filtered

through a short plug of Celite and concentrated *in vacuo*. The crude material was purified on a flash column of silica gel (7:3 CH_2Cl_2:ethyl acetate) to afford 228.5 mg (52%) of **10** as a clear oil. R_f (7:3 CH_2Cl_2:ethyl acetate) = 0.33. ^{1}H NMR (600 MHz, DMSO) δ 2.38 (s, 3H), 2.85 (s, 6H), 3.64–3.67 (m, 2H), 3.68–3.72 (m, 2H), 3.99–4.02 (m, 2H), 4.12–4.15 (m, 2H), 6.74 (dd, J = 7.8, 4.8 Hz, 1H), 7.14 (dd, J = 7.9, 1.4 Hz, 1H), 7.43 (d, J = 8.0 Hz, 2H), 7.74 (dd, J = 4.8, 1.5 Hz, 1H), 7.77 (d, J = 8.3 Hz, 2H). LR-MS calcd for $C_{18}H_{24}N_2O_5S$: m/z 380.1406. Found: $[M+H]^+$ m/z 381.1.

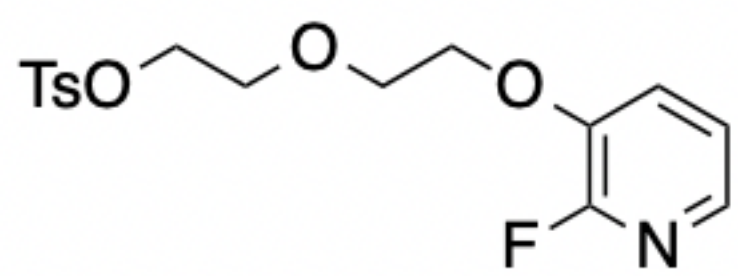

2-(2-((2-Fluoropyridin-3-yl)oxy)ethoxy)ethyl tosylate (11). 2-Fluoropyridin-3-ol (1.025 g, 9.06 mmol) and diethylene glycol di(*p*-toluenesulfonate) (3.670 g, 8.85 mmol) were added to a flame-dried 150 mL round-bottom flask, under Ar. The compounds were dissolved in dry MeCN (50 mL) and powdered K_2CO_3 (2.445 g, 17.69 mmol) was added. The mixture was refluxed under Ar for 2.5 h, then cooled to room temperature, quenched with ethyl acetate (60 mL) and stirred for an additional 10 min. The reaction was then filtered and concentrated *in vacuo*. The crude material was purified on a flash column of silica gel (9:1 CH_2Cl_2–ethyl acetate, then 4:1 CH_2Cl_2:ethyl acetate, then 100% ethyl acetate) to afford 1.128 g (38%) of 11 as a yellow-orange oil. R_f (9:1 CH_2Cl_2:ethyl acetate) = 0.54. ^{1}H NMR (600 MHz, DMSO) δ 2.38 (s, 3H), 3.65 (s, 2H), 3.70 (s, 2H), 4.15 (s, 4H), 7.29 (d, J = 3.1 Hz, 1H), 7.44 (d, J = 8.1 Hz, 2H), 7.63 (s, 1H), 7.73 (s, 1H), 7.77 (d, J = 8.4 Hz, 2H). ^{19}F NMR (471 MHz, DMSO) δ -85.36. LR-MS calcd for $C_{16}H_{18}FNO_5S$: m/z 356.089. Found: $[M]^+$ m/z 356.1.

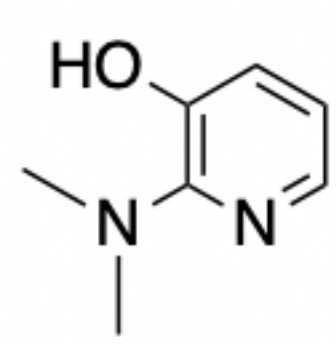

2-Dimethylamino-3-hydroxypyridine (12). This procedure was based on the preparation of 2-dimethylamino-3-(2-(2-(prop-2-ynyloxy)ethoxy)ethoxy)pyridine from Inkster *et al.*,[2] except MeOH was used as the solvent instead of water. 2-Fluoro-3-hydroxypyridine (750 mg, 6.63 mmol) was dissolved in a

solution of 2 M trimethylamine in MeOH (49.7 mL, 99.48 mmol, 15 eq). The mixture was refluxed for 20 h, cooled to room temperature and concentrated by rotary evaporation. The resulting crude product was purified twice by silica gel column chromatography, first with 1:2 hexanes:ethyl acetate, then with 1:1 hexanes:ethyl acetate, to afford **12** (160 mg, 17%) as a white solid. *Rf* (1:1 hexanes:ethyl acetate) = 0.24. ^{1}H NMR (600 MHz, $CDCl_3$) δ 2.75 (s, 6H), 6.96 (dd, J = 7.9, 4.8 Hz, 1H), 7.16 (dd, J = 7.8, 1.6 Hz, 1H), 7.93 (dd, J = 4.8, 1.6 Hz, 1H). LR-MS calcd for $C_7H_{10}N_2O$: m/z 138.170. Found: $[M+H]^+$ m/z 139.1.

References

(1) Vasdev, N.; Collier, T. L. Design and Prototype of an Automated Column-Switching HPLC System for Radiometabolite Analysis. *Pharmaceuticals* **2016**, *9* (3), 51. https://doi.org/10.3390/ph9030051.

(2) Inkster, J.; Lin, K.-S.; Ait-Mohand, S.; Gosselin, S.; Bénard, F.; Guérin, B.; Pourghiasian, M.; Ruth, T.; Schaffer, P.; Storr, T. 2-Fluoropyridine Prosthetic Compounds for the ^{18}F Labeling of Bombesin Analogues. *Bioorg. Med. Chem. Lett.* **2013**, *23* (13), 3920–3926. https://doi.org/10.1016/j.bmcl.2013.04.060.

Additional Figures (S1 - S5) and Table (S1)

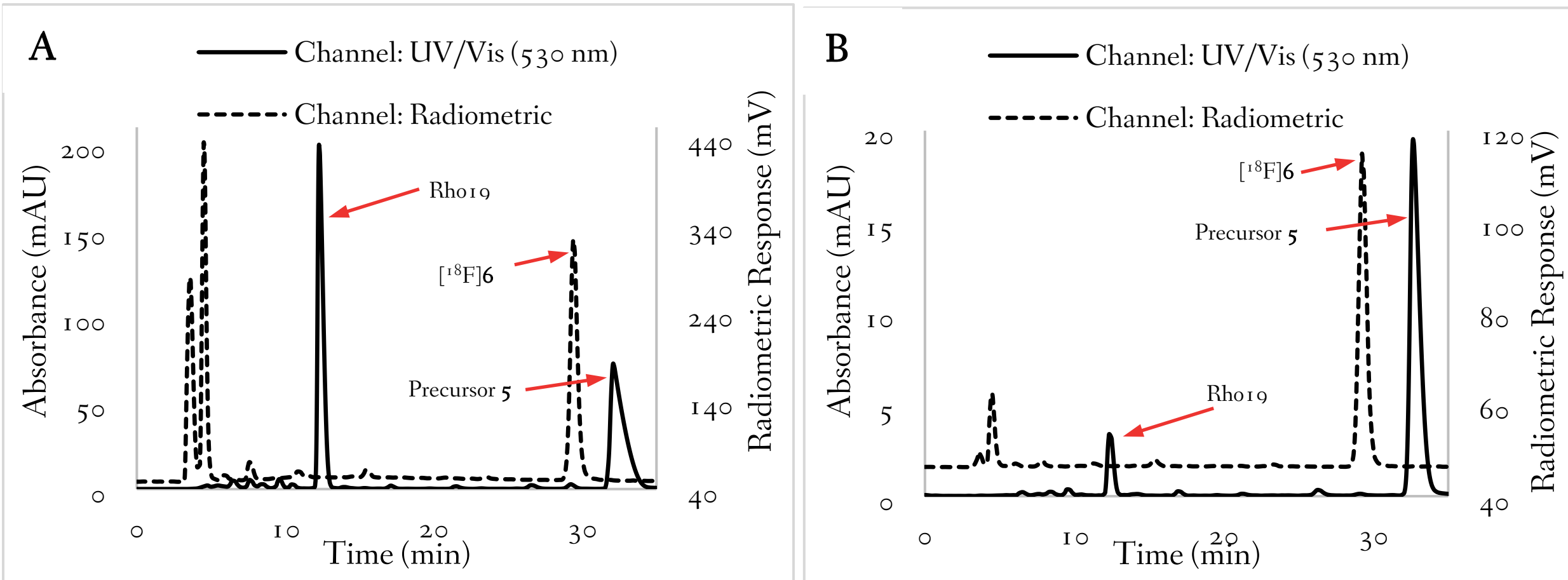

Figure S1. Crude traces of the semi-prep C18-HPLC isolation of radiotracer [^{18}F]**6**. **HPLC A, Program B** was used. **A)** Run 5. **B)** Run 11 (See **Table 1** in the main text). Significant degradation of precursor **5** to Rhodamine 19 is seen when K_2CO_3 was used as anion exchanger (Run 5), which was mitigated by switching to $TEAHCO_3$ (Run 11).

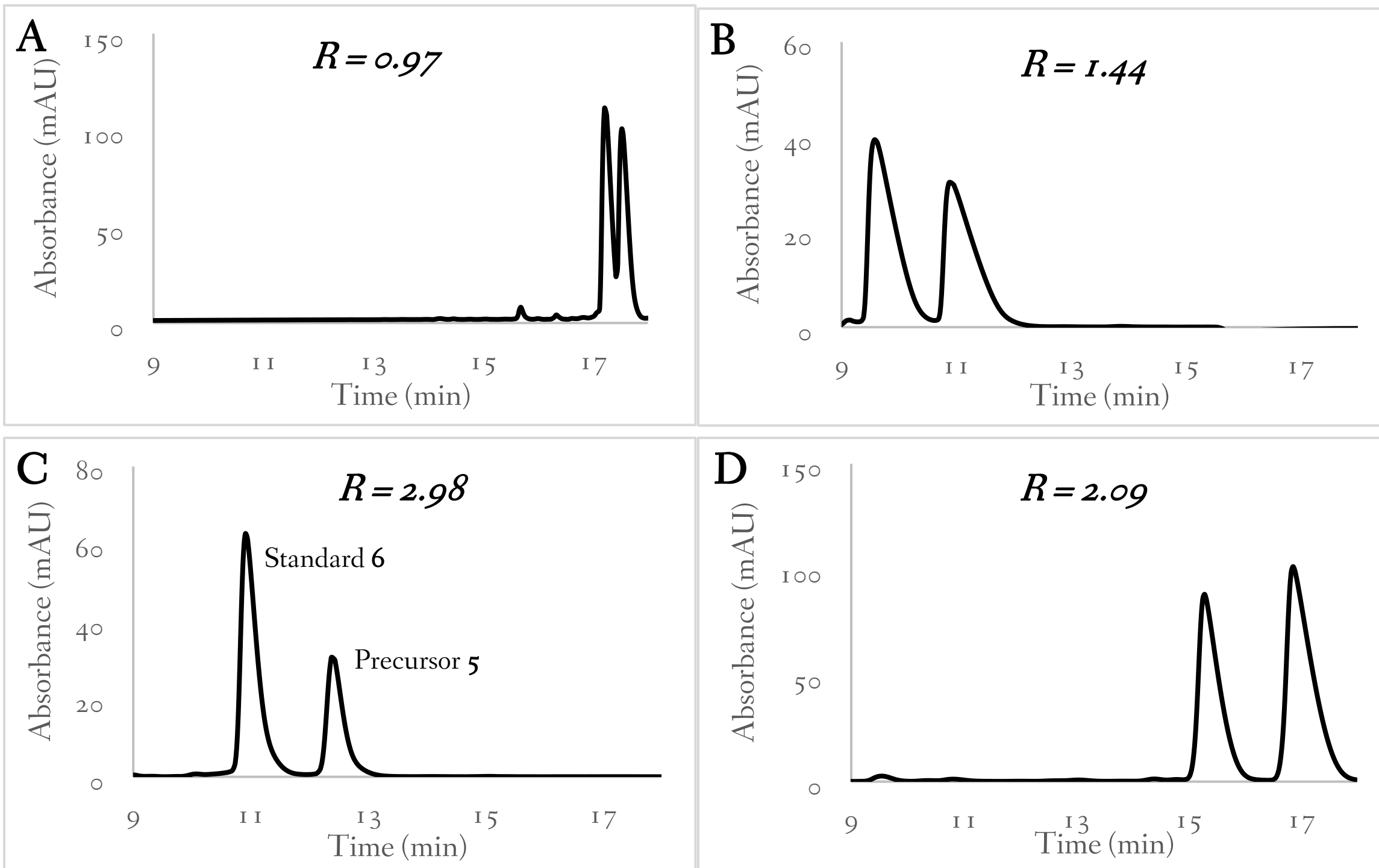

Figure S2. HPLC separation (HPLC A) of compounds **5** and **6** using C18 PFP columns ~~(HPLC A)~~, with peak resolution values (R) shown on each trace. Traces A, B and C were acquired using an analytical column, while Trace D was acquired using a semi-preparative column. Compounds **5** and **6** elute in the same order ~~with each program~~in each trace. A) Gradient elution; 10% MeCN in H_2O containing 0.1% formic acid for 2 min, raised to 70% over 18 min, raised to 95% over 5 min (**Program G**). B) Isocratic elution; 45:55 MeCN:H_2O containing 0.1% formic acid (**Program H**). C) Isocratic elution; 45:55 MeCN:H_2O containing 0.1% TFA (**Program I**). D) Isocratic elution; 50:50 MeCN:H_2O containing 0.1% TFA (**Program A**).

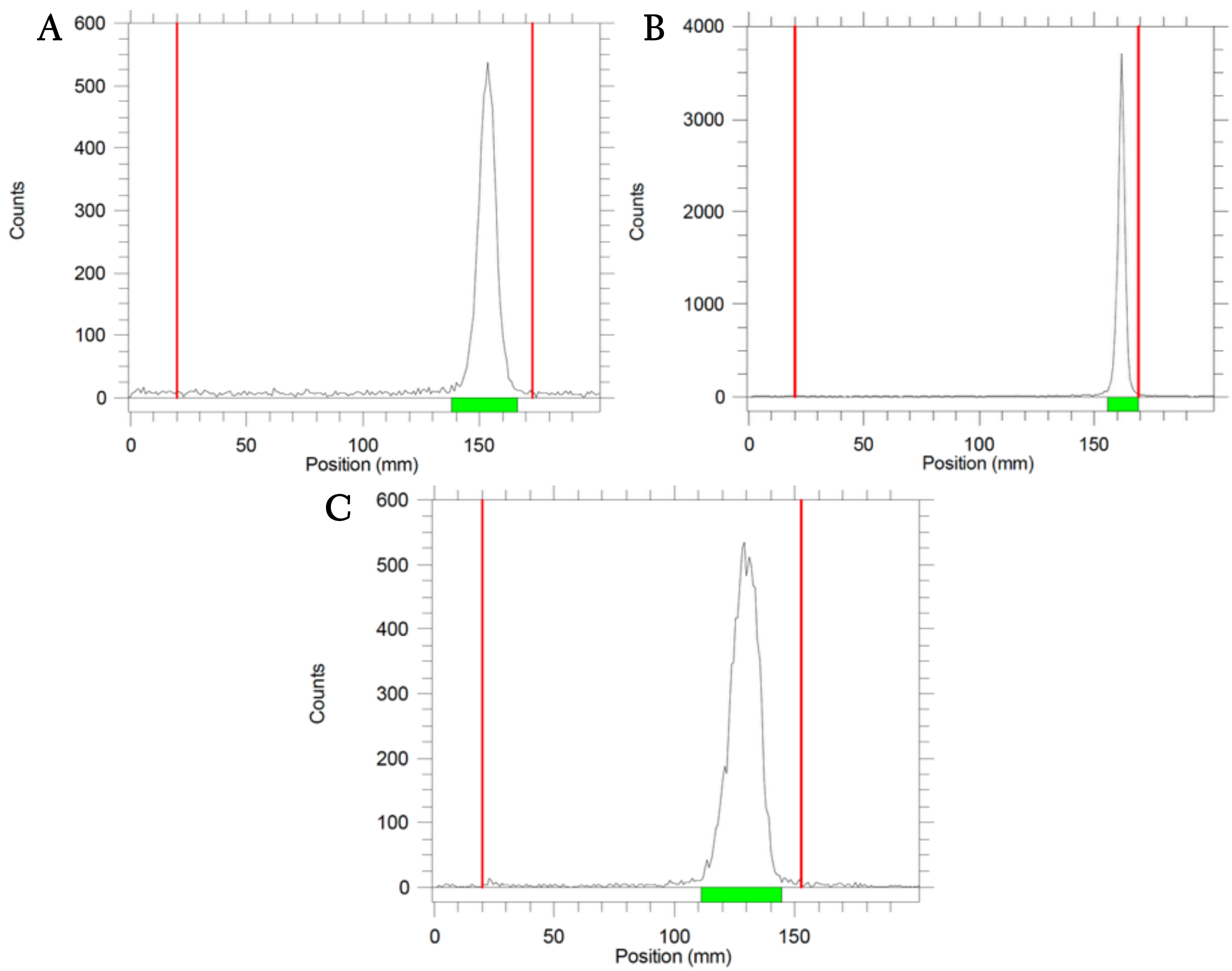

Figure S3. Radio-TLC traces from stability evaluation of [^{18}F]**6** in A) PBS (1×, pH 7.4), B) rat serum, and C) mouse serum at 120 minutes [iTLC-SA plates (silicic acid, Molpure, Inc), elution with 20% MeOH in CH_2Cl_2]. Retention factors vary slightly due to batch-to-batch variability of TLC matrix, which was made separately for each experiment and maintained for several hours over the course of each radiosynthesis and serum incubation period. (see **Table S1** below).

Table S1. Average retention factors (*n*=3) for all time points assayed in serum stability studies.

Timepoint	PBS control	Rat serum	Mouse serum
15 min	0.91±0.02	0.94±0.01	0.72±0.01
60 min	0.89±0.01	0.95±<0.01	0.75±0.03
120 min	0.84±0.03	0.95±0.01	0.80±0.01

[^{18}F]**6** appears >99% intact in every trace measured.

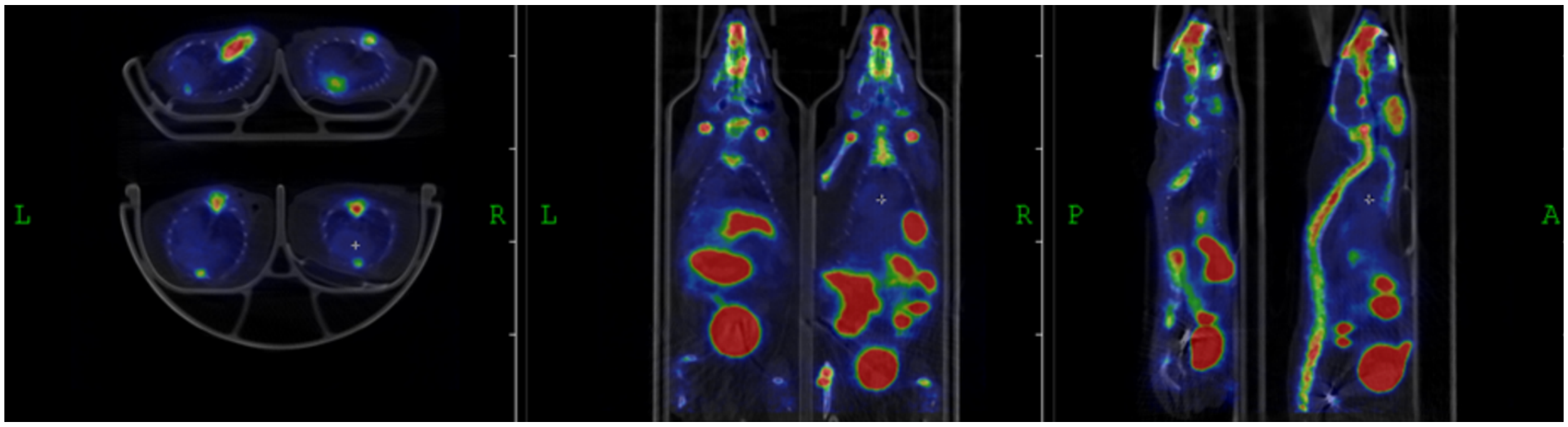

Figure S4. Micro-PET images of [^{18}F]Rho6G-DEG-Py-F ([^{18}F]**6**) in mice. Images are composite over 90-120 min. Left: transverse plane; Middle: coronal plane; Right: sagittal plane. [^{18}F]Rho6G-DEG-Py-F is rapidly metabolized in this species; further details can be found in the main text.

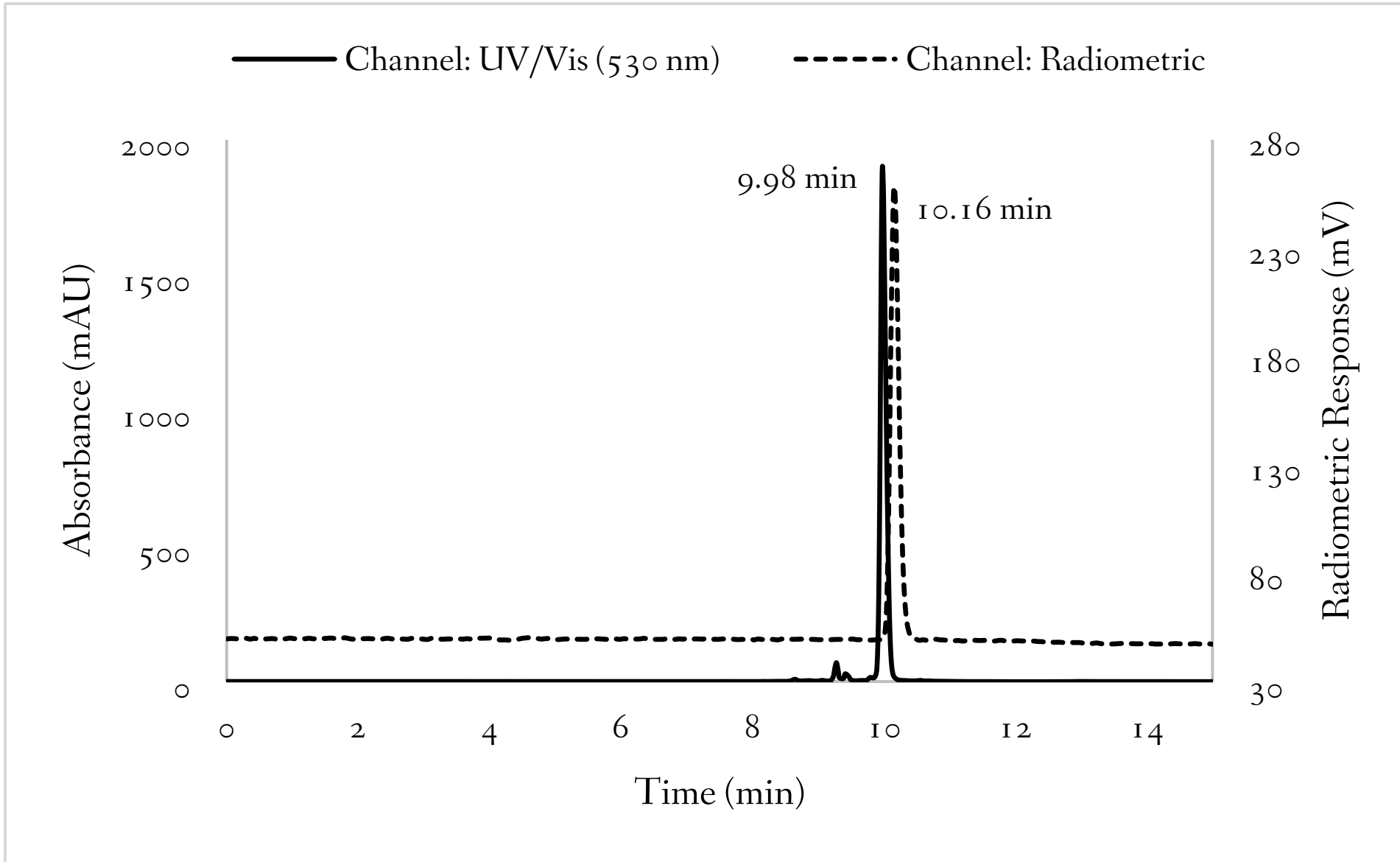

Figure S5. HPLC (**HPLC A, Program C**) trace of the co-injection of **6** and [^{18}F]**6** (**Table 1**, Run 9). Retention times are slightly staggered because the isotopologues pass through the UV detector first.

NMR and ESI Mass Spectra of Non-radioactive Compounds

Rhodamine 6G lactone (4)

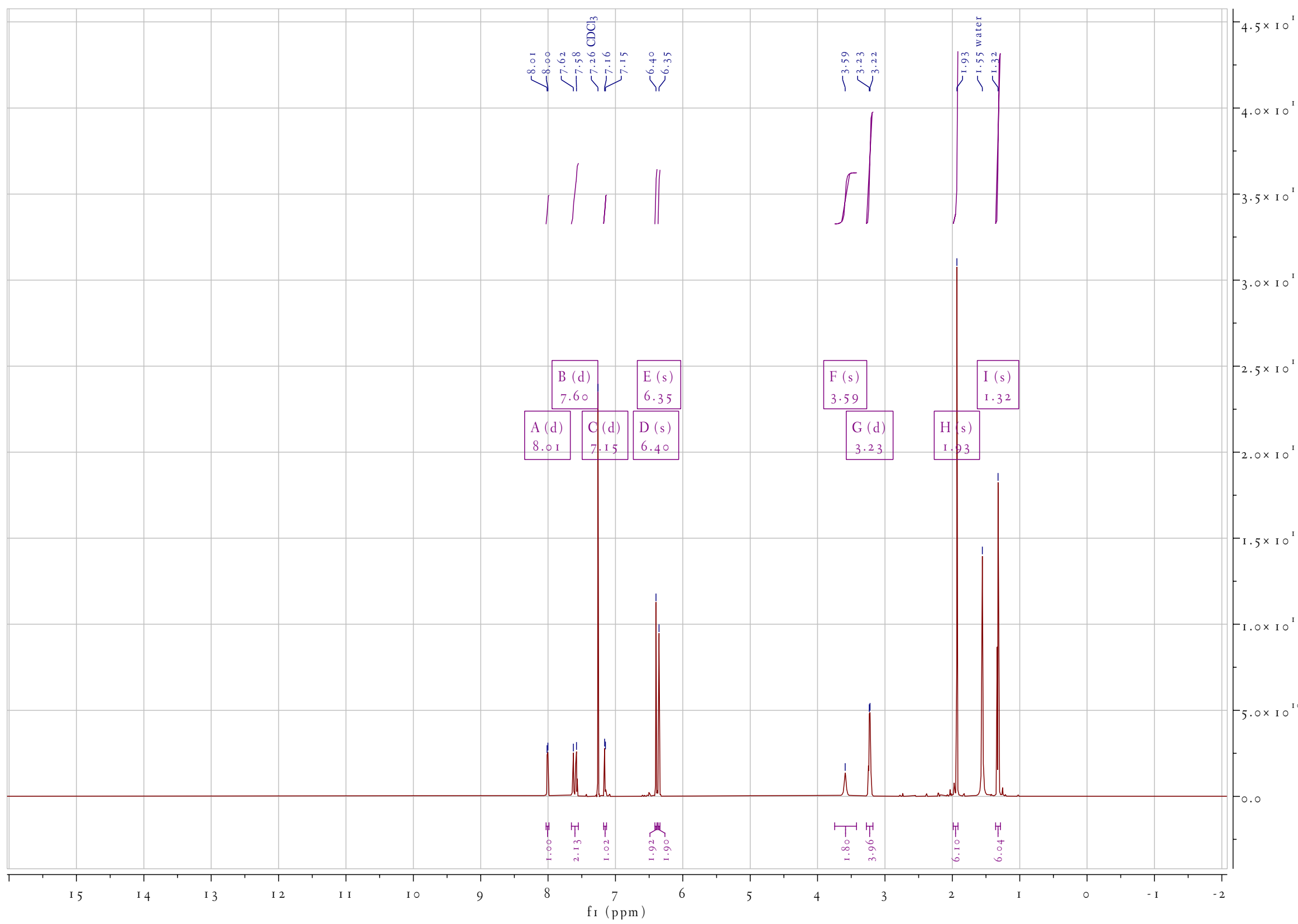

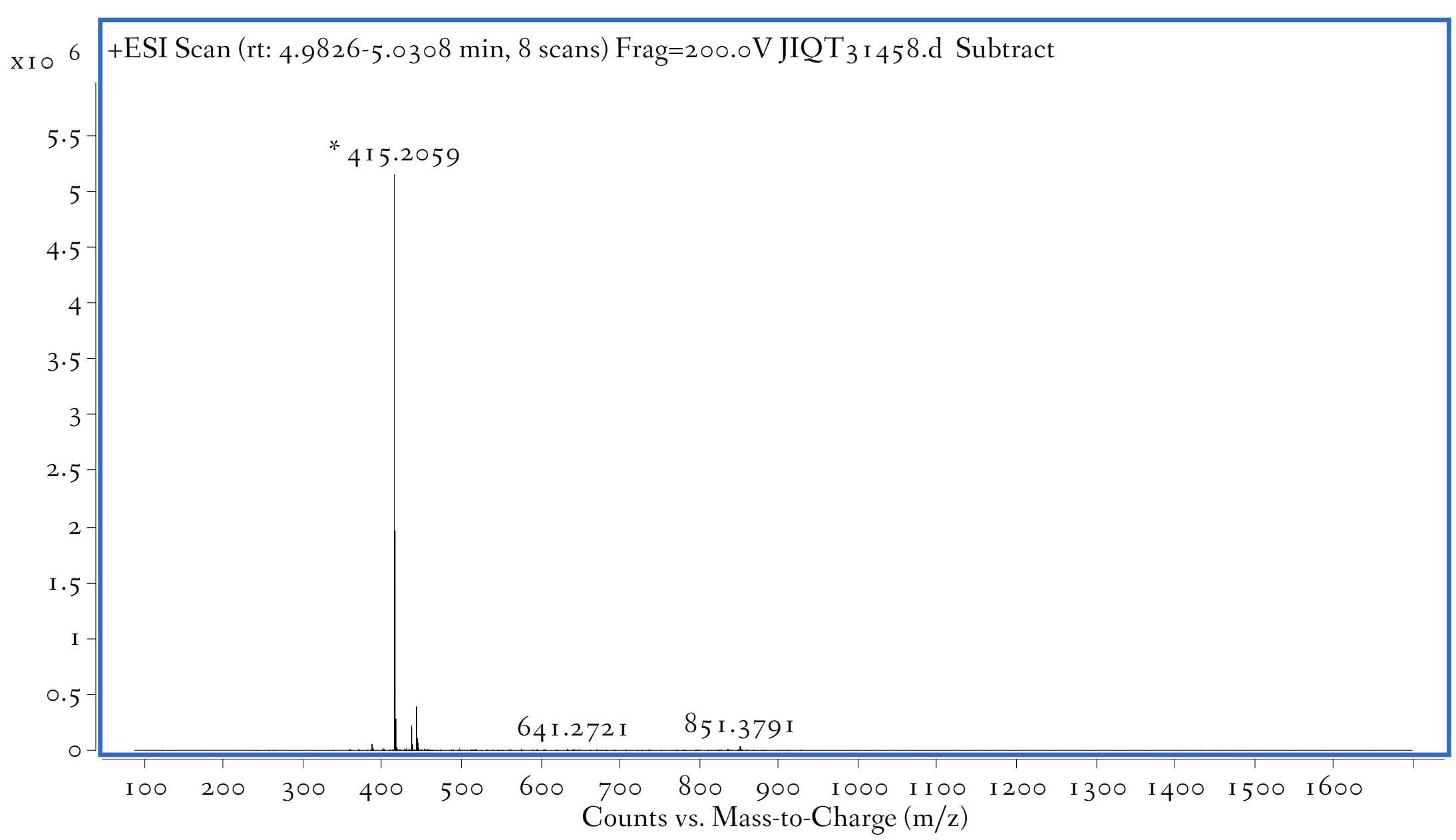

2-(2-((2-Fluoropyridin-3-yl)oxy)ethoxy)ethyl tosylate (11)

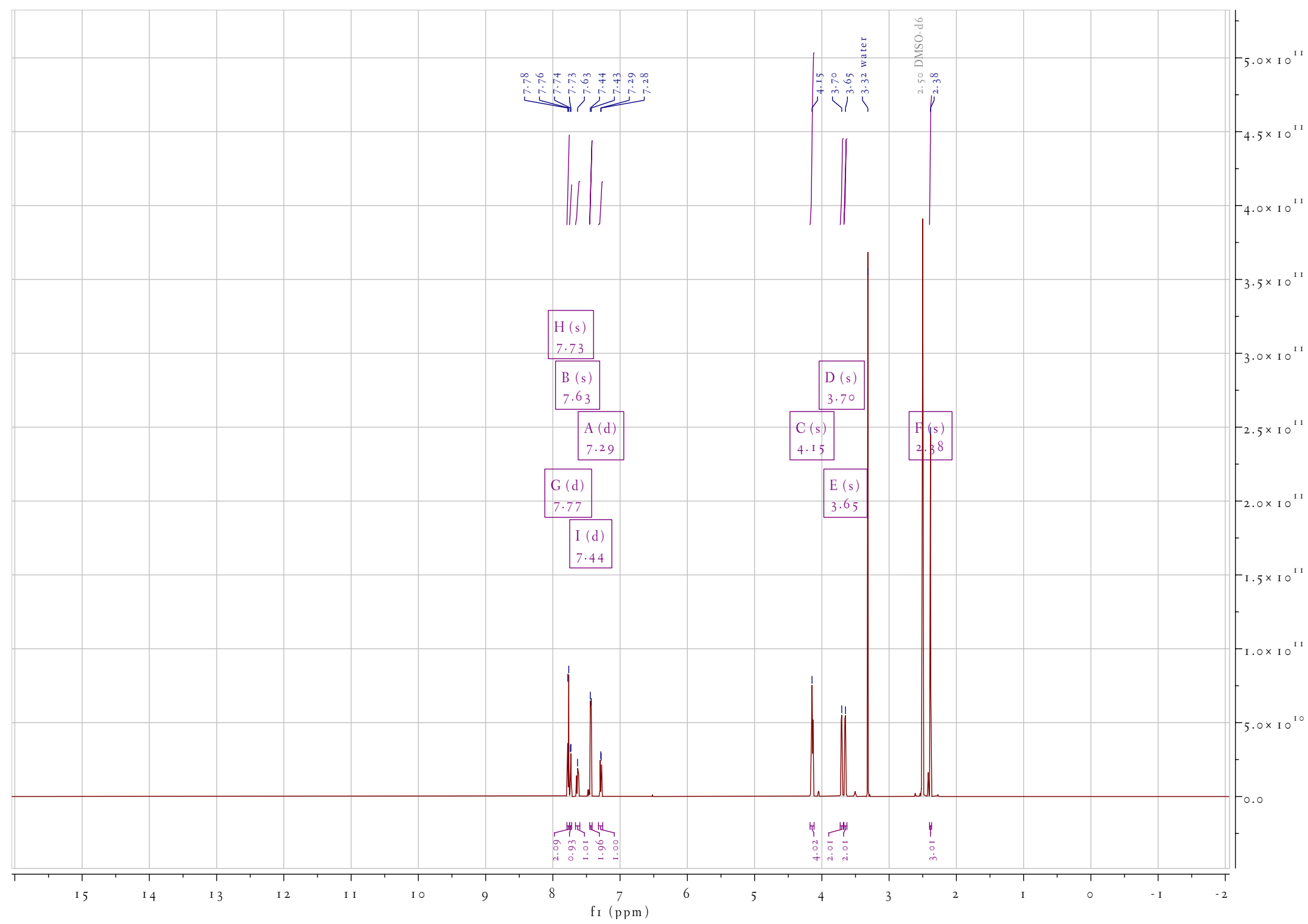

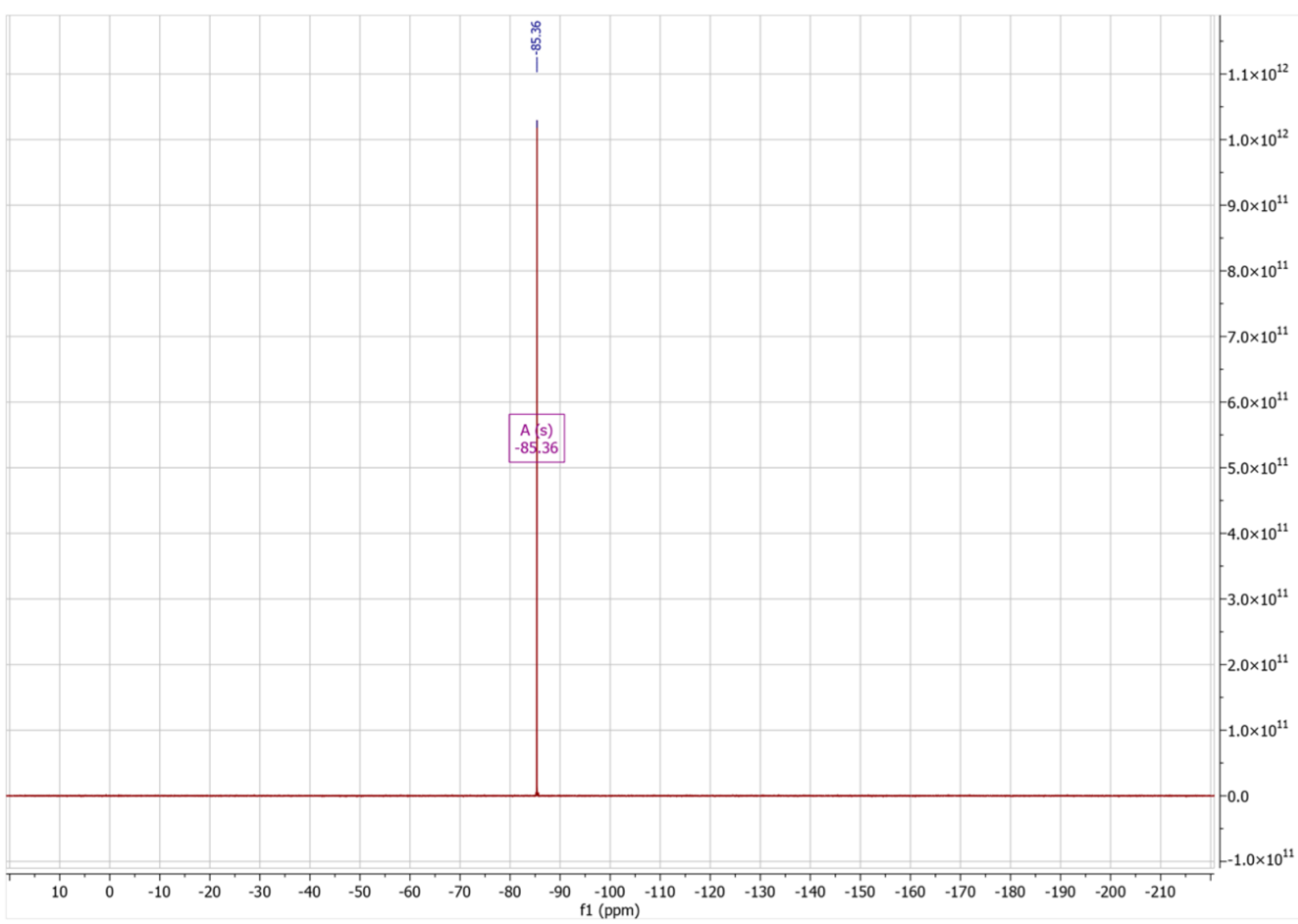

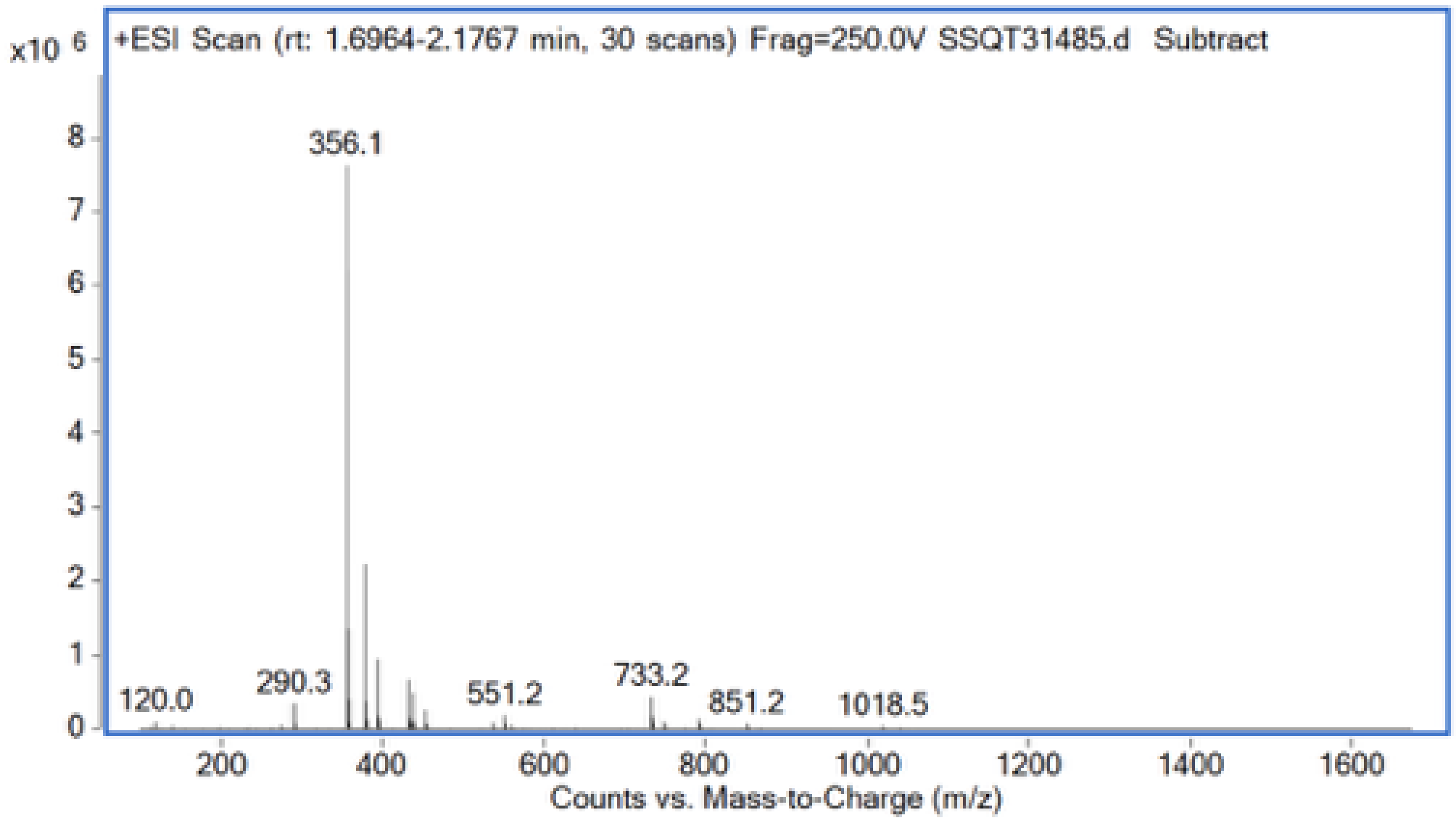
x10 6
+ESI Scan (rt: 1.6964-2.1767 min, 30 scans) Frag=250.0V SSQT31485.d Subtract
356.1
120.0
290.3
551.2
733.2
851.2
1018.5
Counts vs. Mass-to-Charge (m/z)

Rho6G-DEG-Py-F (6)

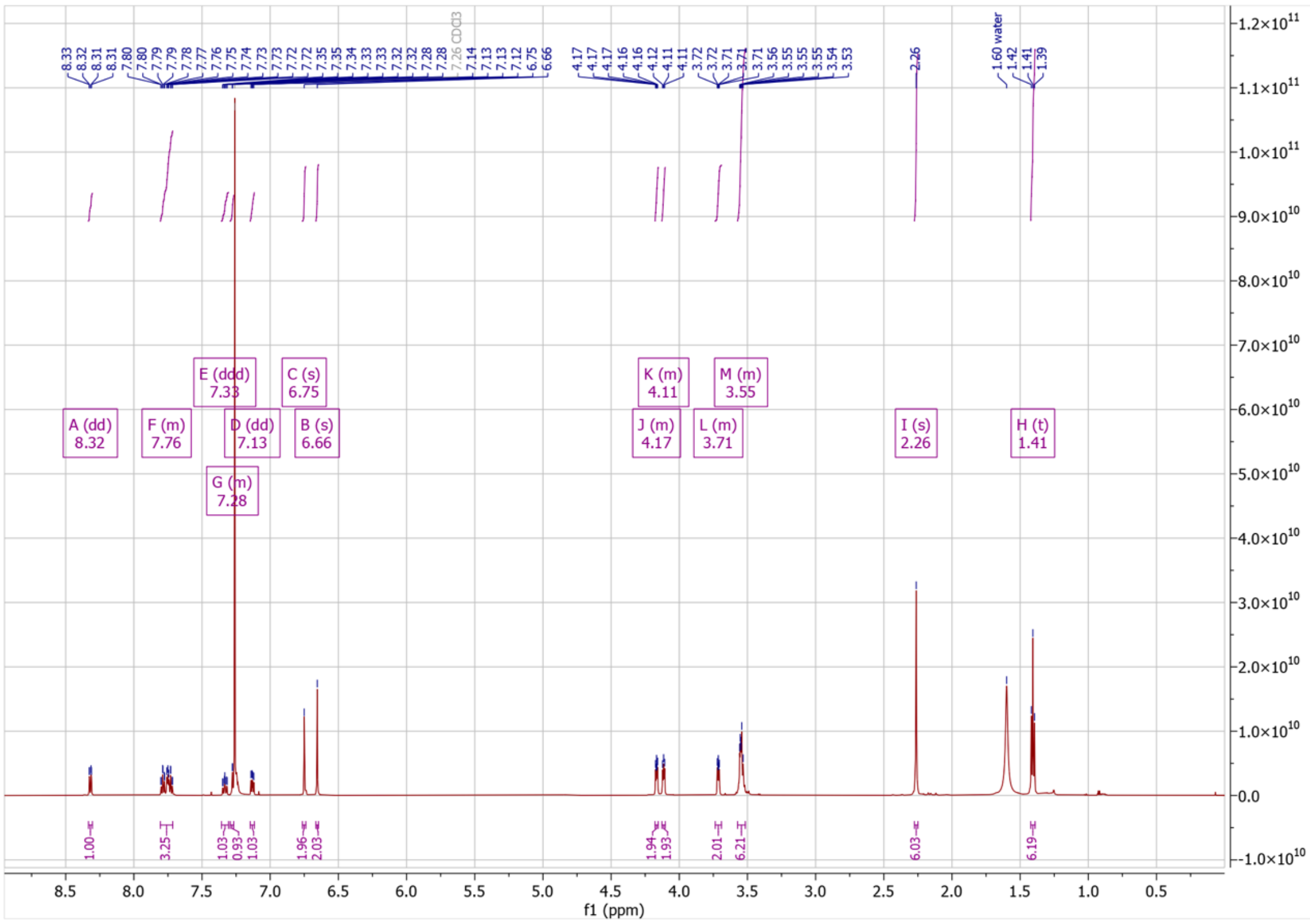

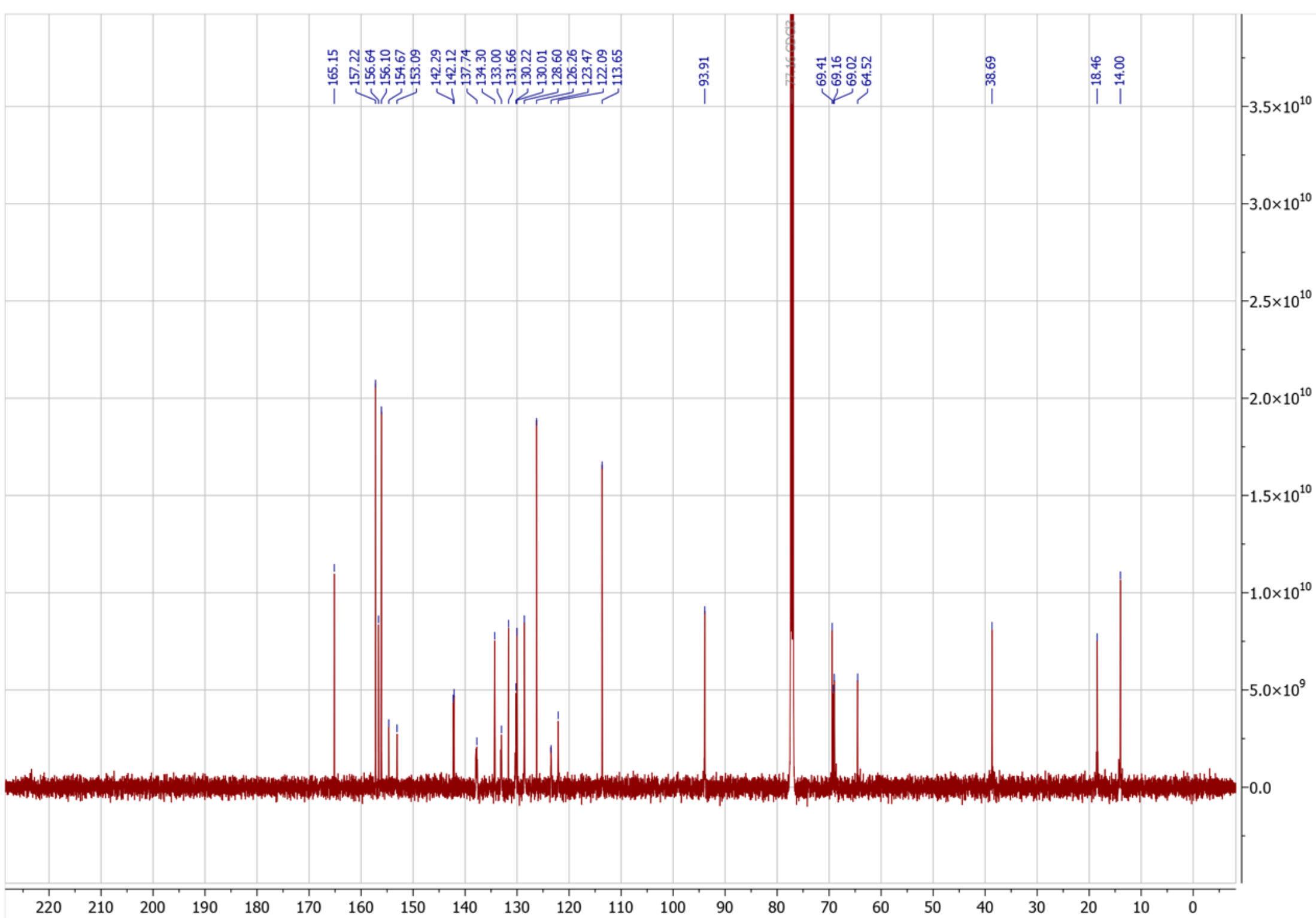

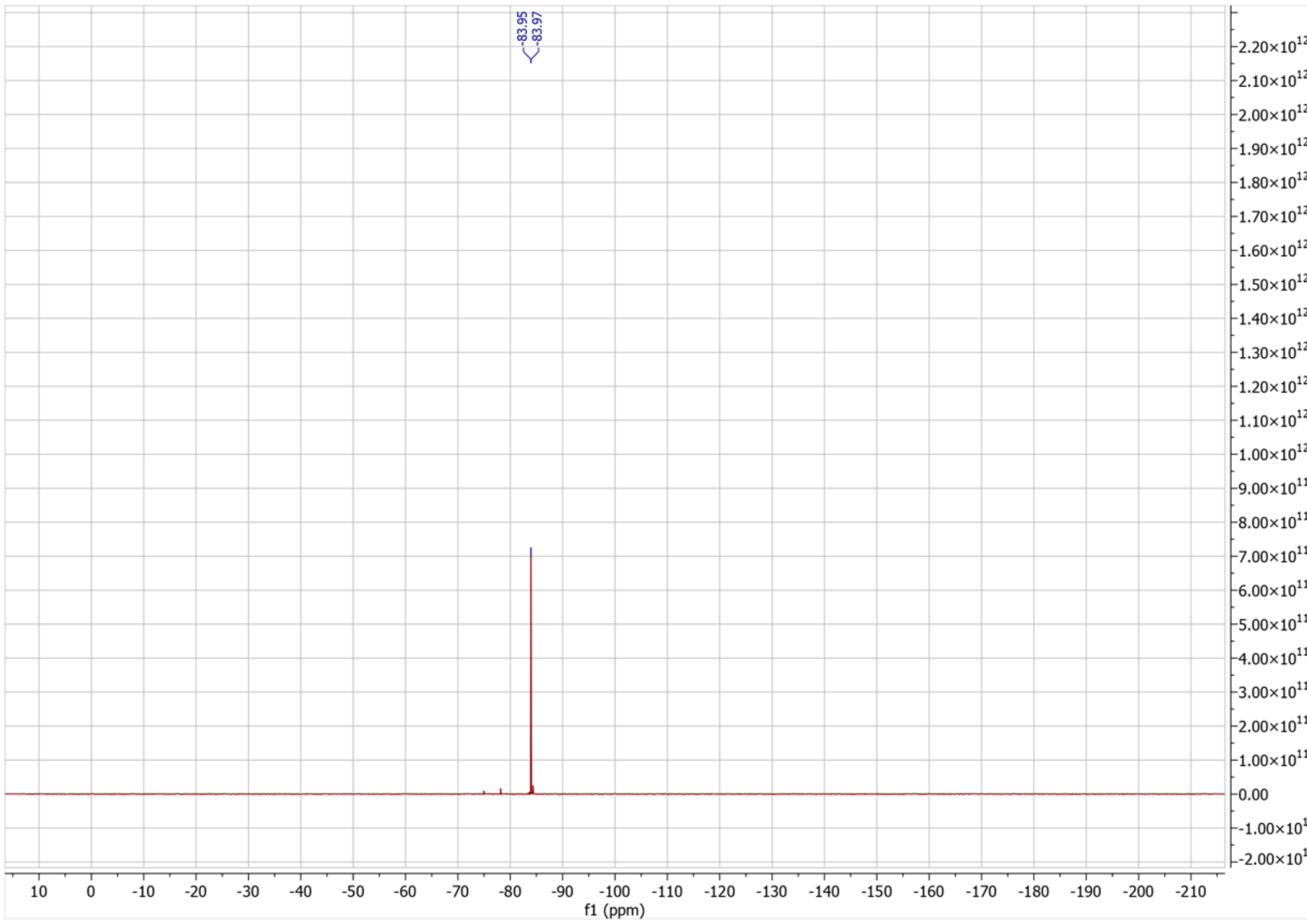

-83.95
-83.97
f1 (ppm)

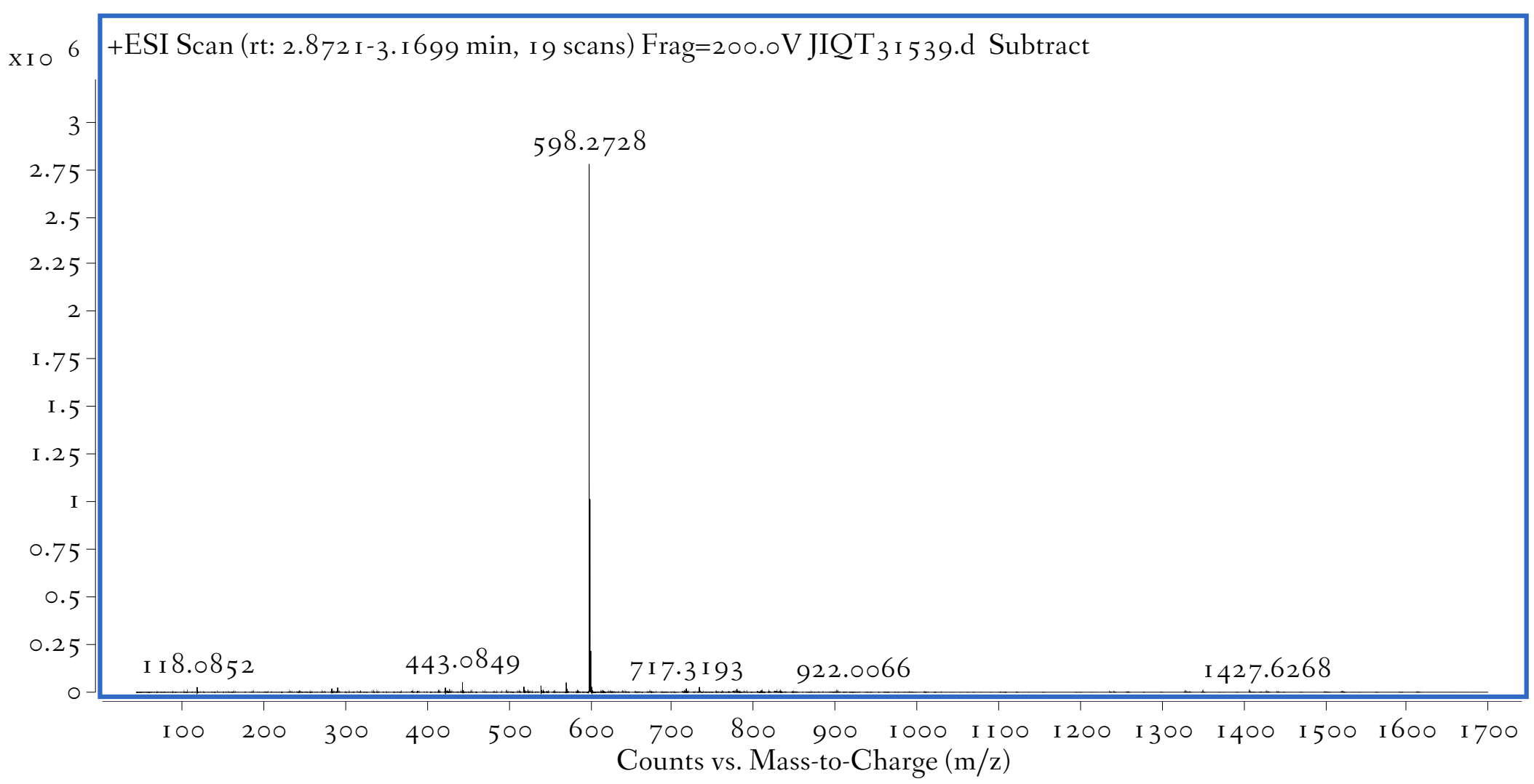

x10 6
+ESI Scan (rt: 2.8721-3.1699 min, 19 scans) Frag=200.0V JIQT31539.d Subtract
598.2728
118.0852
443.0849
717.3193
922.0066
1427.6268
Counts vs. Mass-to-Charge (m/z)

2-(2-(2-nitropyridin-3-yloxy)ethoxy)ethyl tosylate (9)

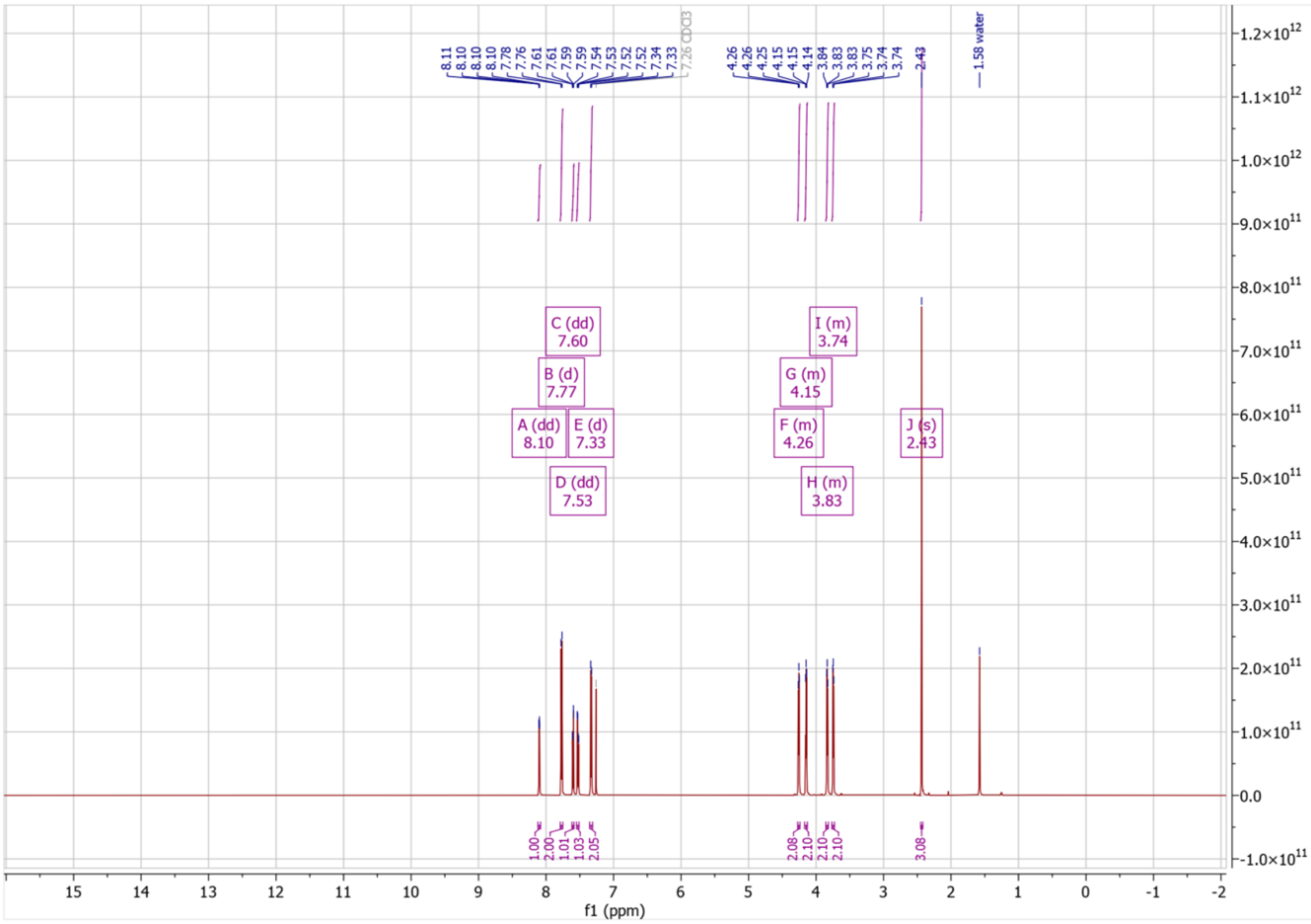

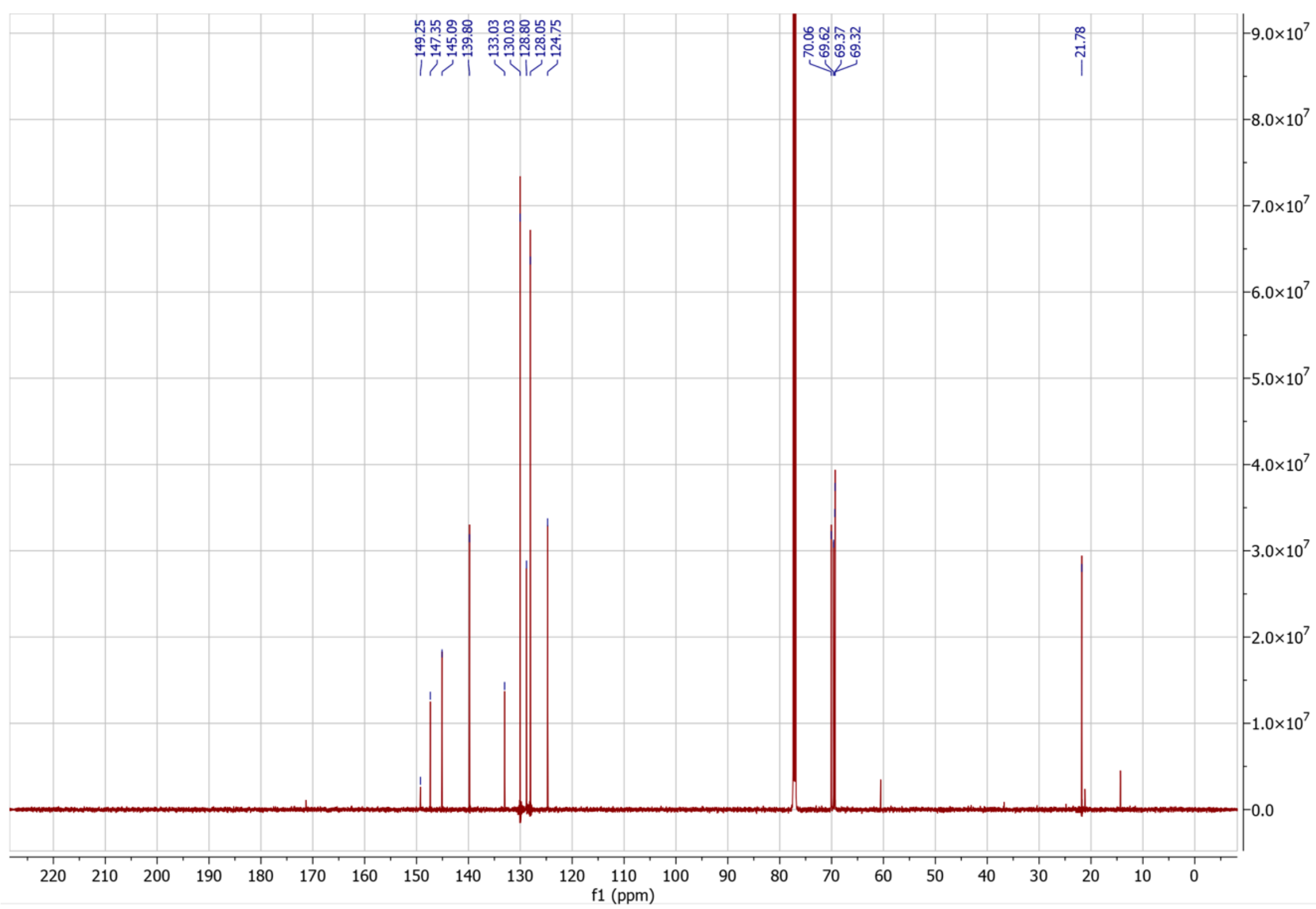

Rho6G-DEG-Py-NO_2 (5)

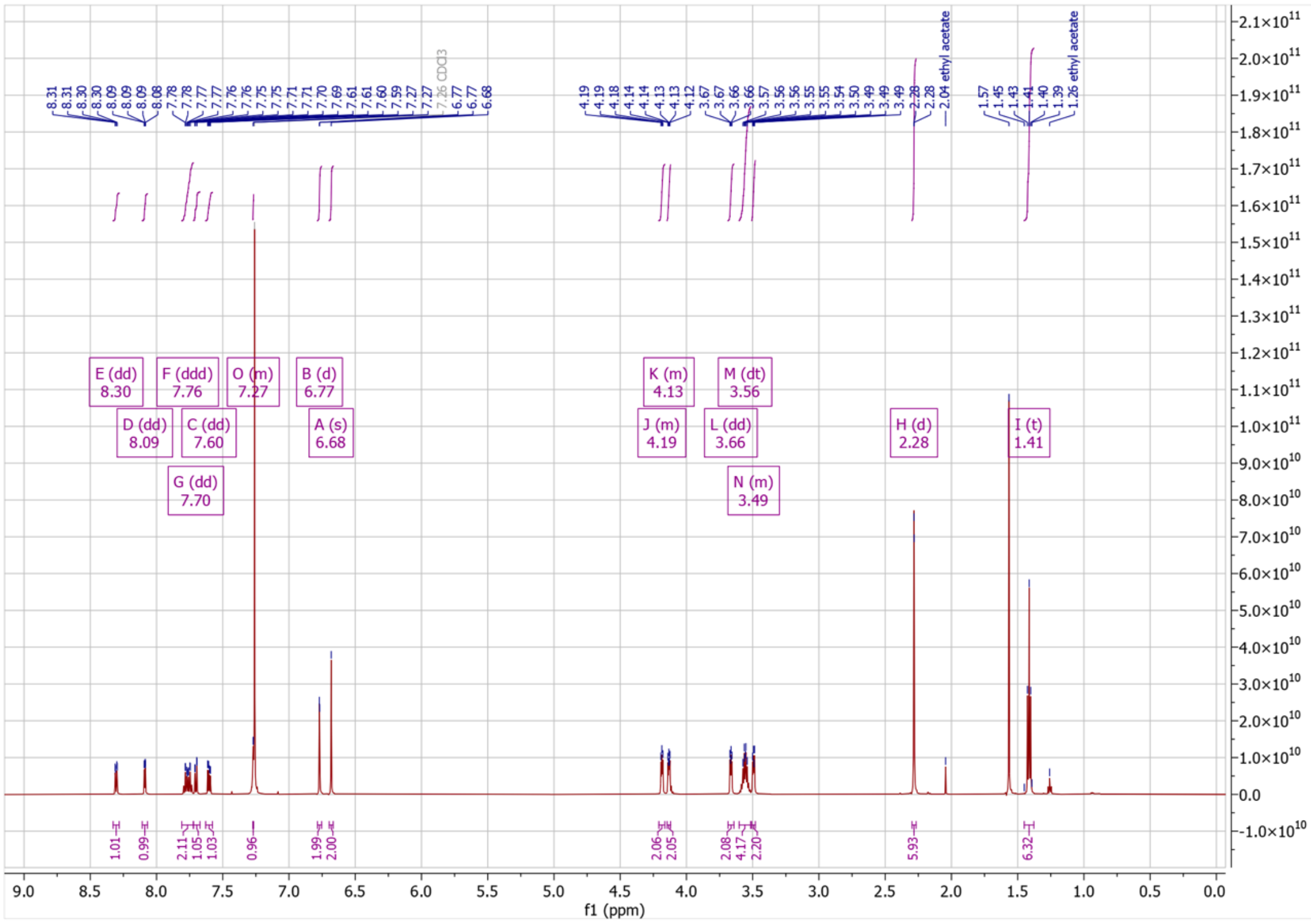

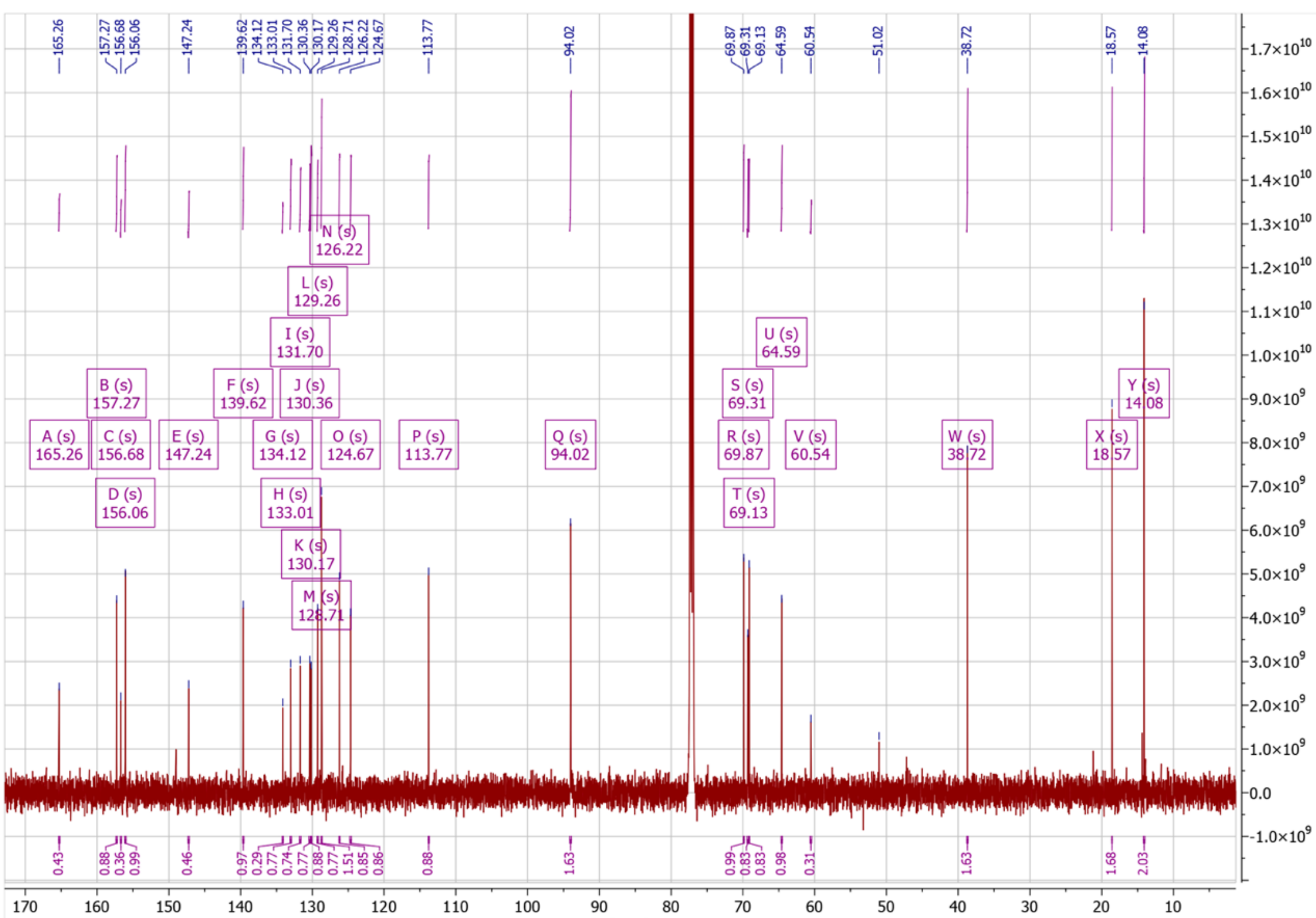

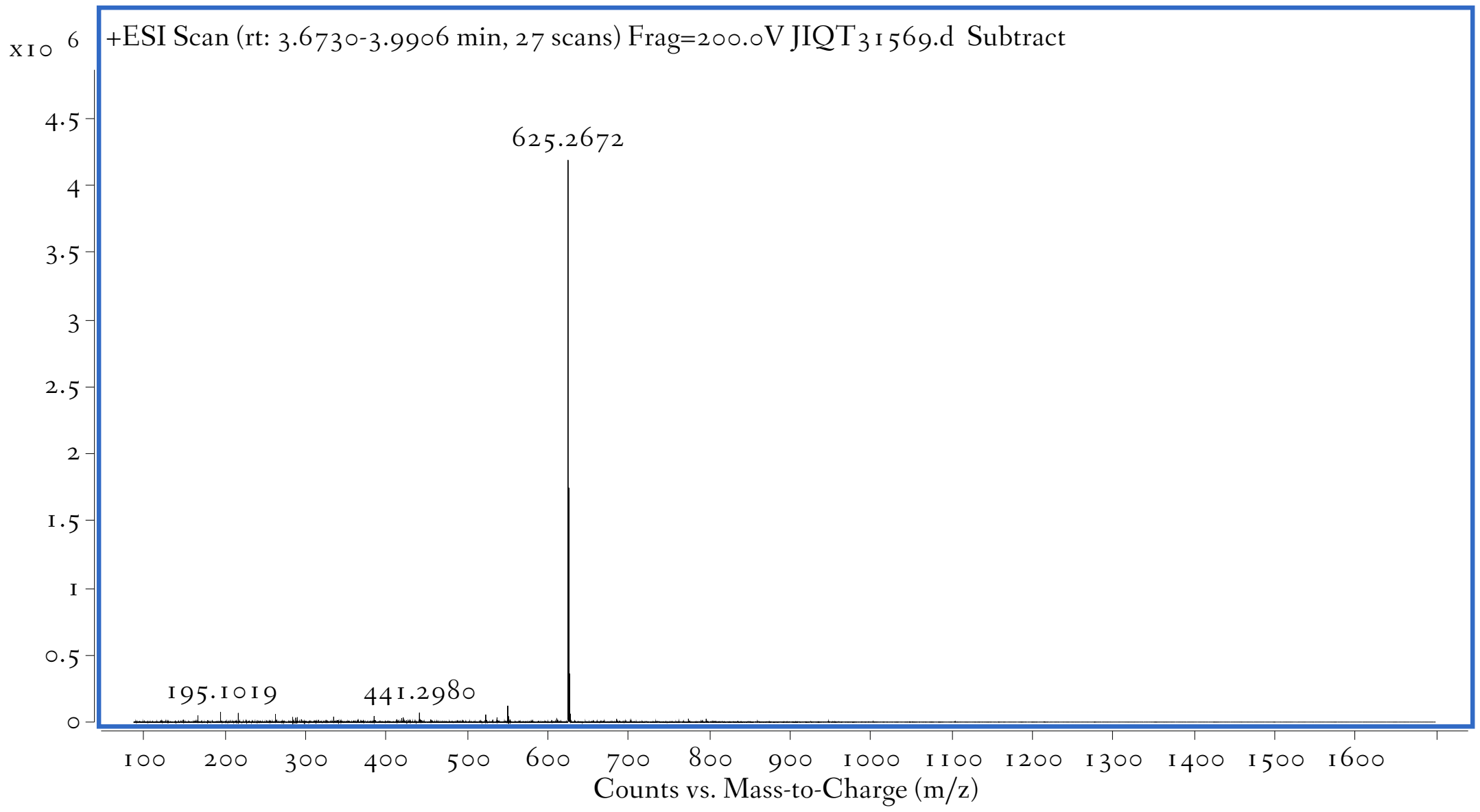
x10 6
+ESI Scan (rt: 3.6730-3.9906 min, 27 scans) Frag=200.0V JIQT31569.d Subtract
625.2672
195.1019
441.2980
0
0.5
1
1.5
2
2.5
3
3.5
4
4.5
100 200 300 400 500 600 700 800 900 1000 1100 1200 1300 1400 1500 1600
Counts vs. Mass-to-Charge (m/z)

2-dimethylamino-3-hydroxypyridine (12)

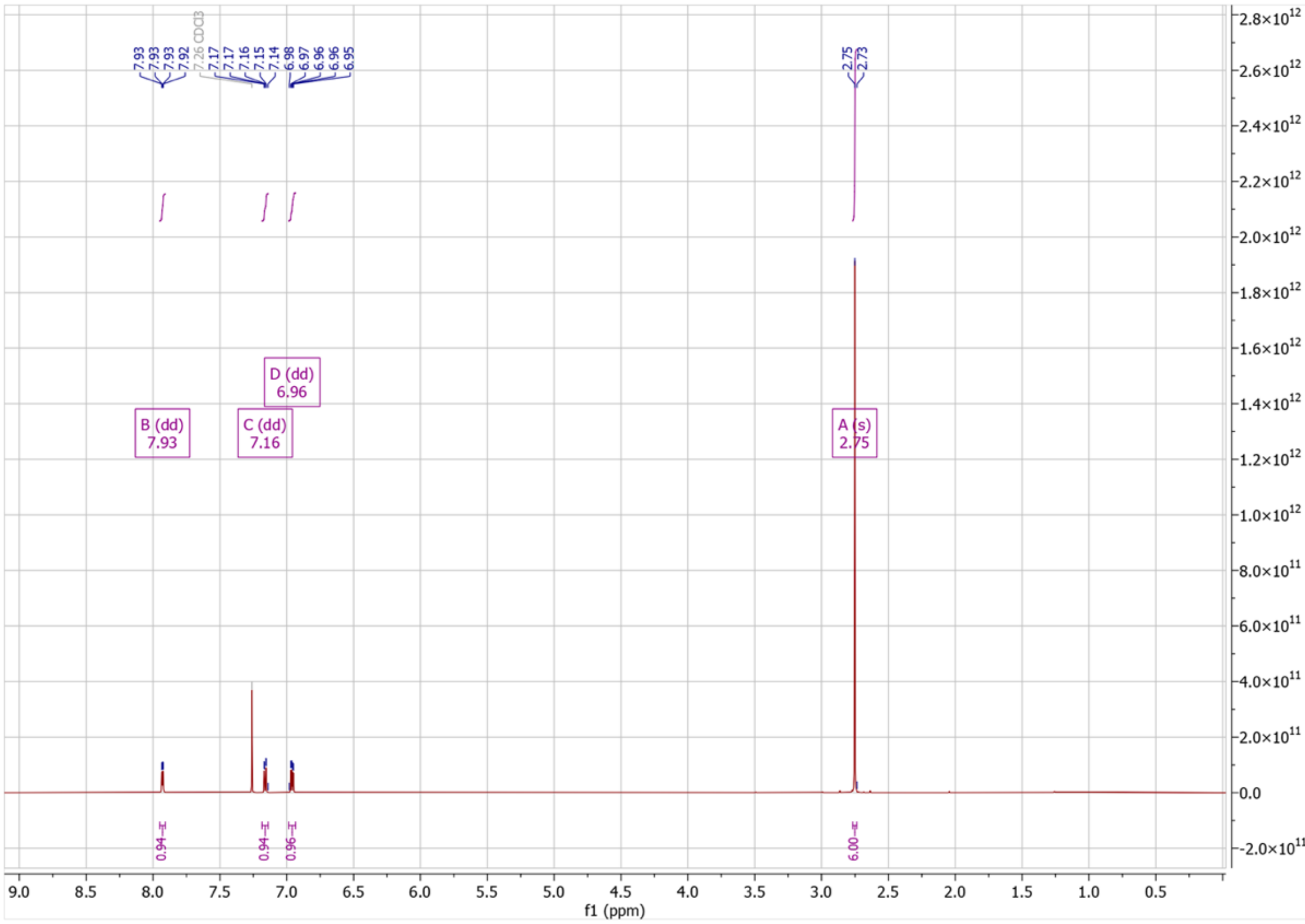

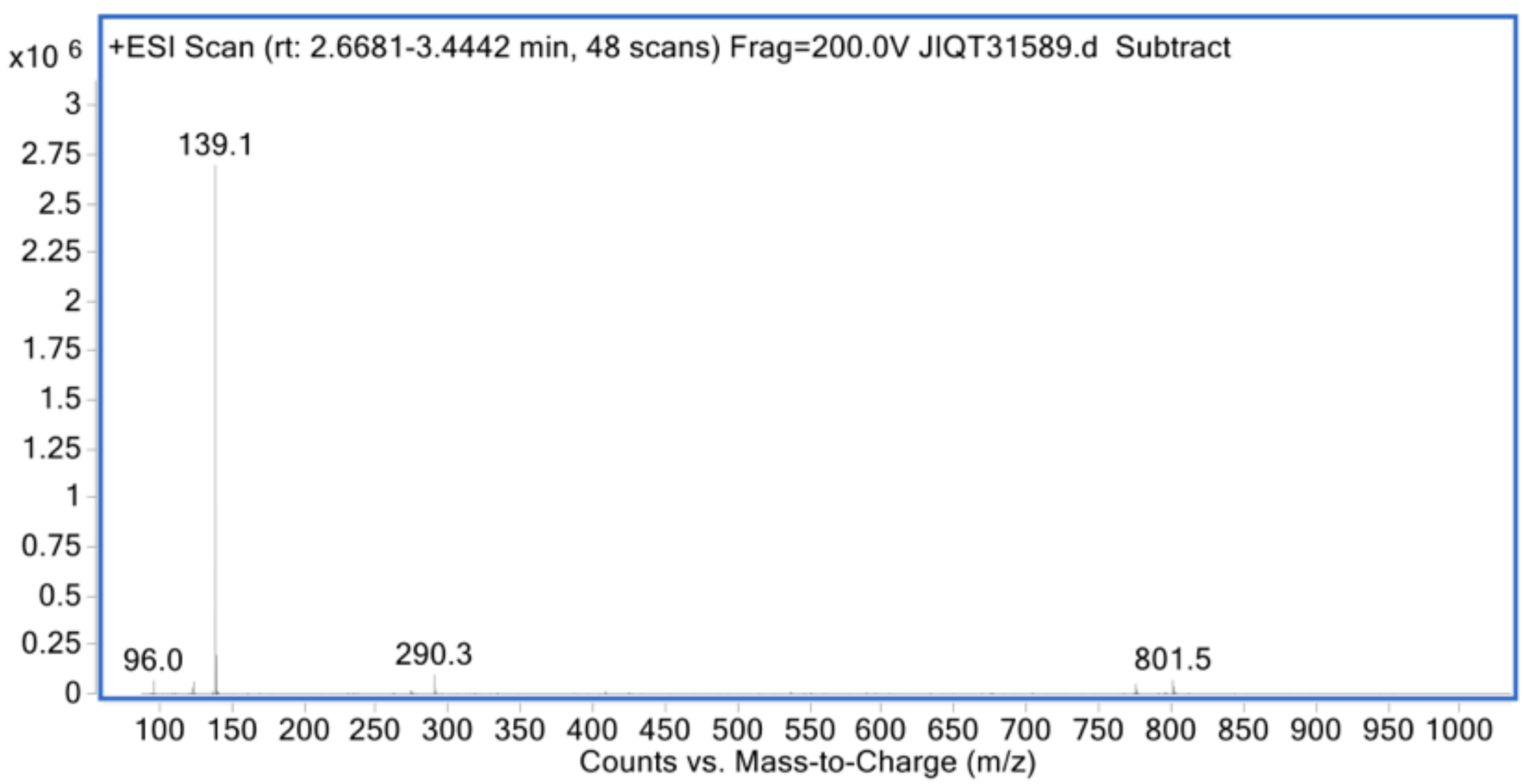

2-(2-((2-dimethylaminopyridin-3-yl)oxy)ethoxy)ethyl tosylate (10)

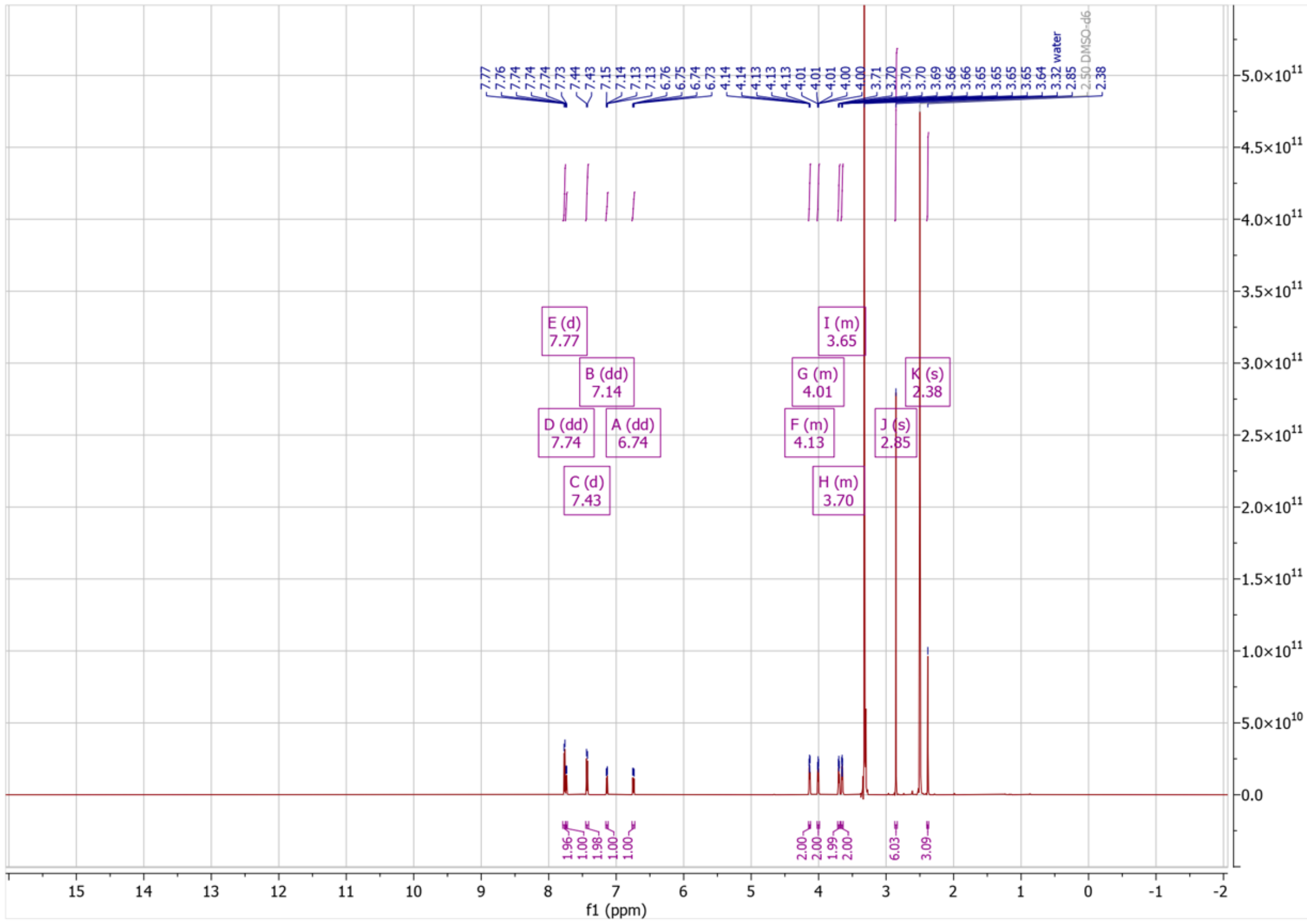

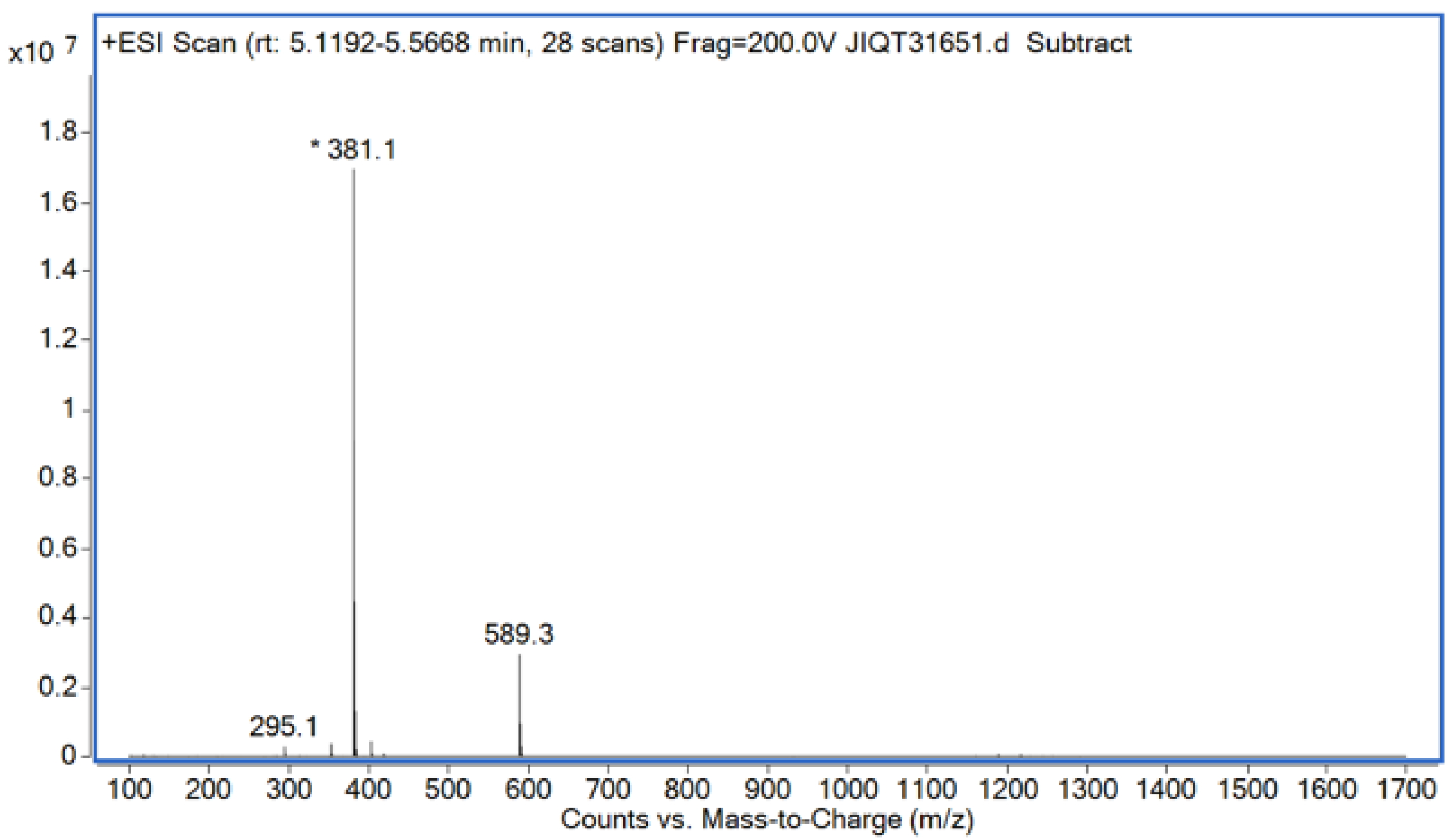

Rho6G-DEG-Py-NMe_2 (7)

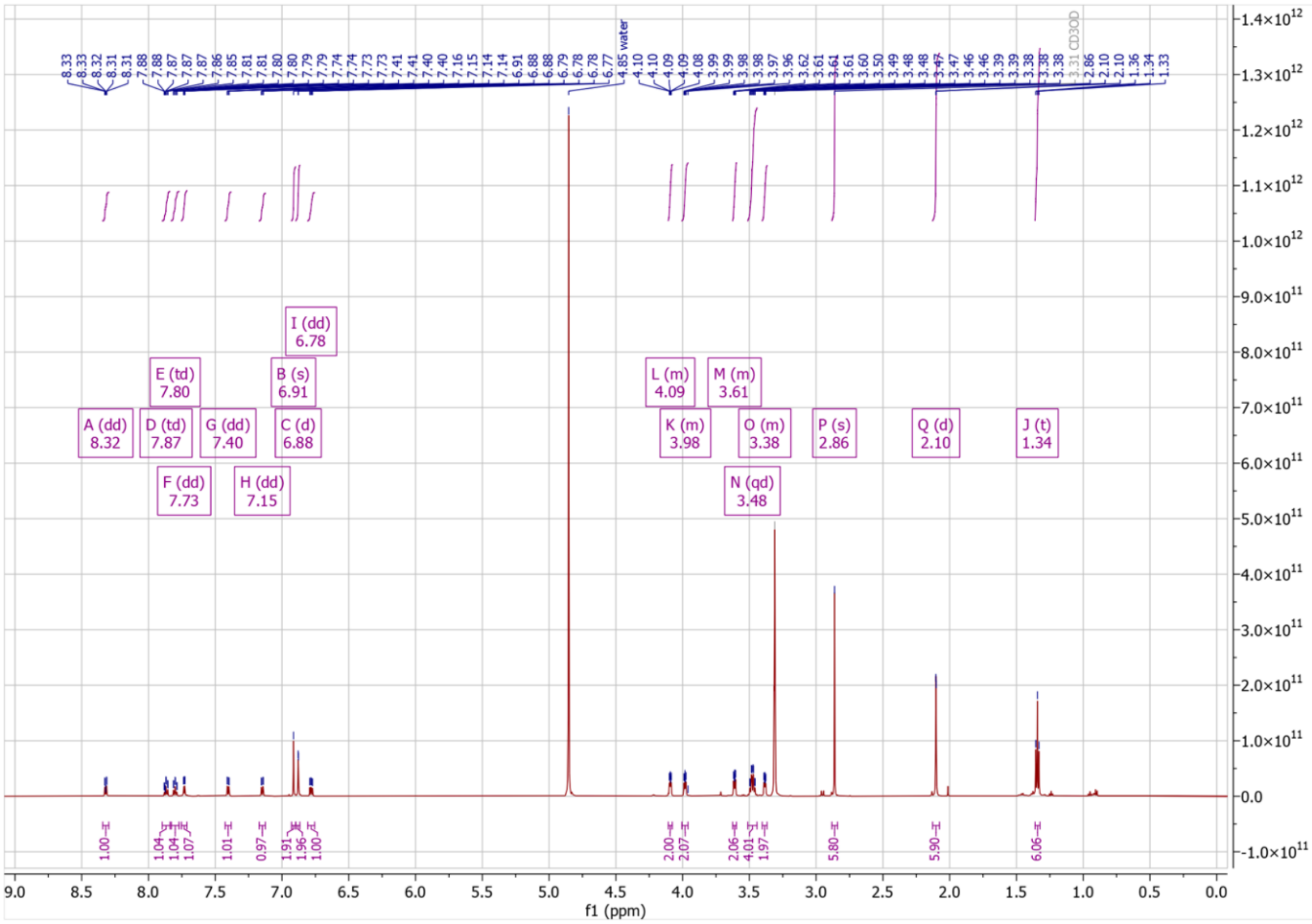

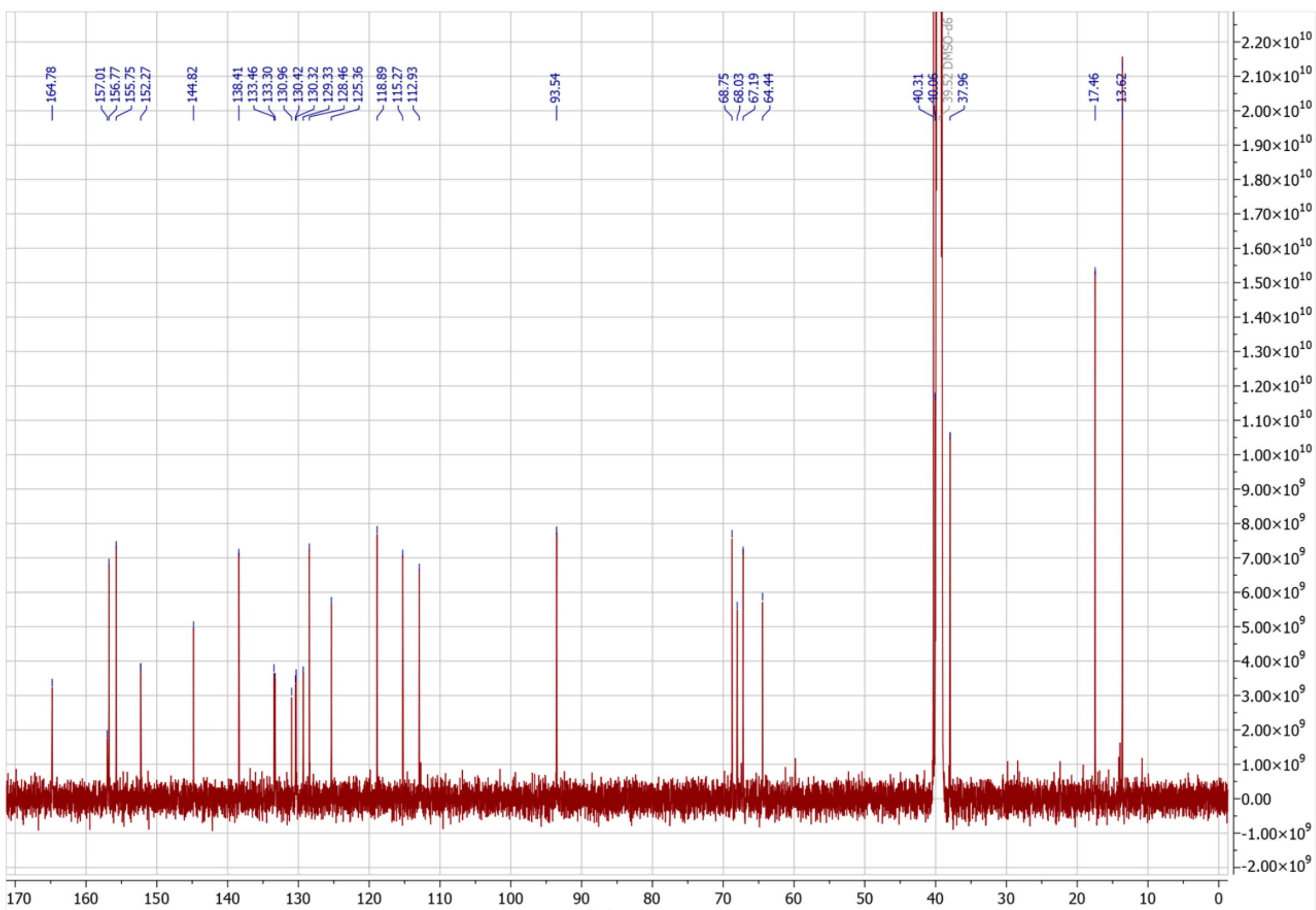

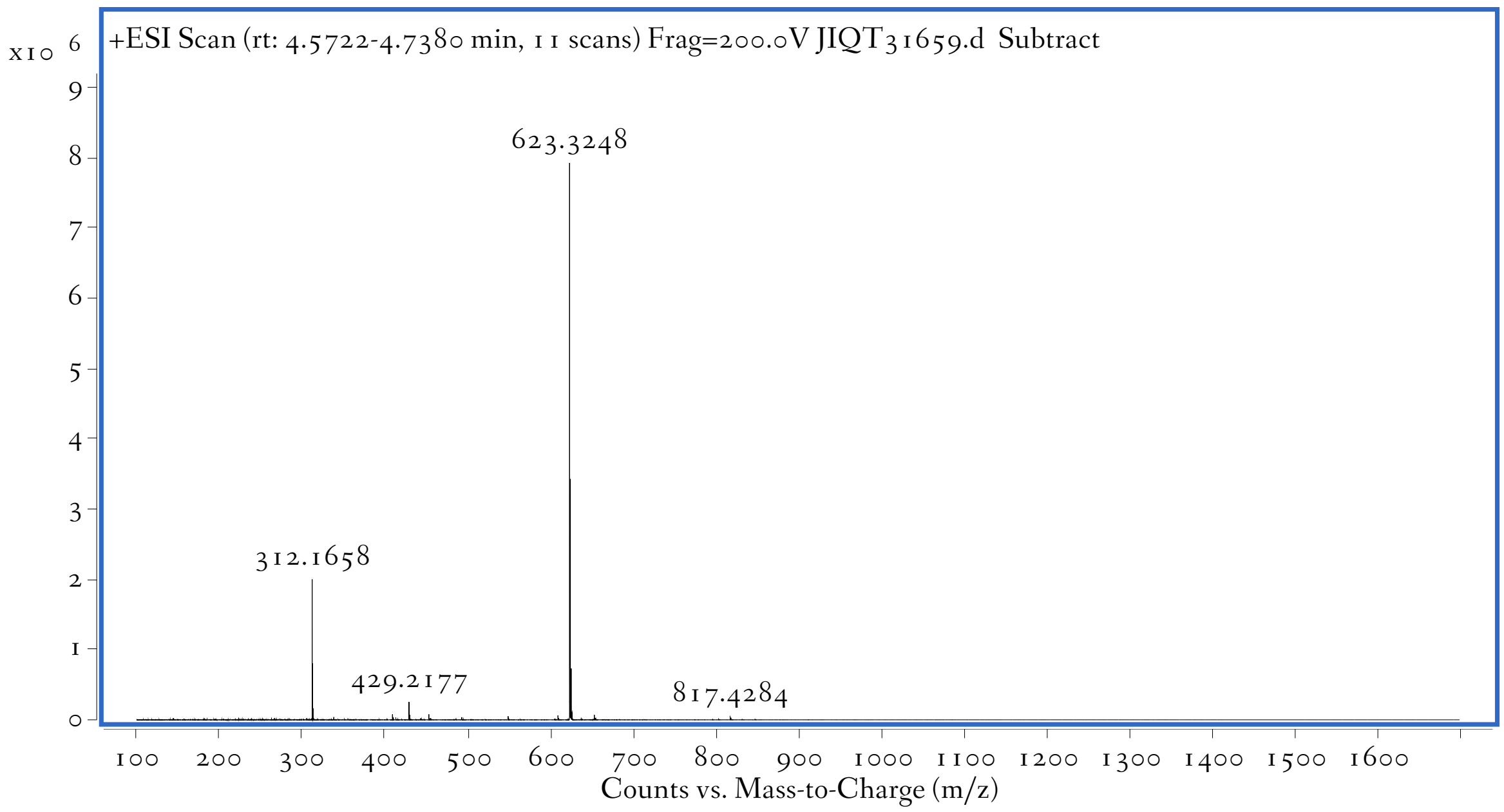
x10 6
+ESI Scan (rt: 4.5722-4.7380 min, 11 scans) Frag=200.0V JIQT31659.d Subtract
623.3248
312.1658
429.2177
817.4284
Counts vs. Mass-to-Charge (m/z)

Rho6G-DEG-Py-NMe$_3^+$ (8)

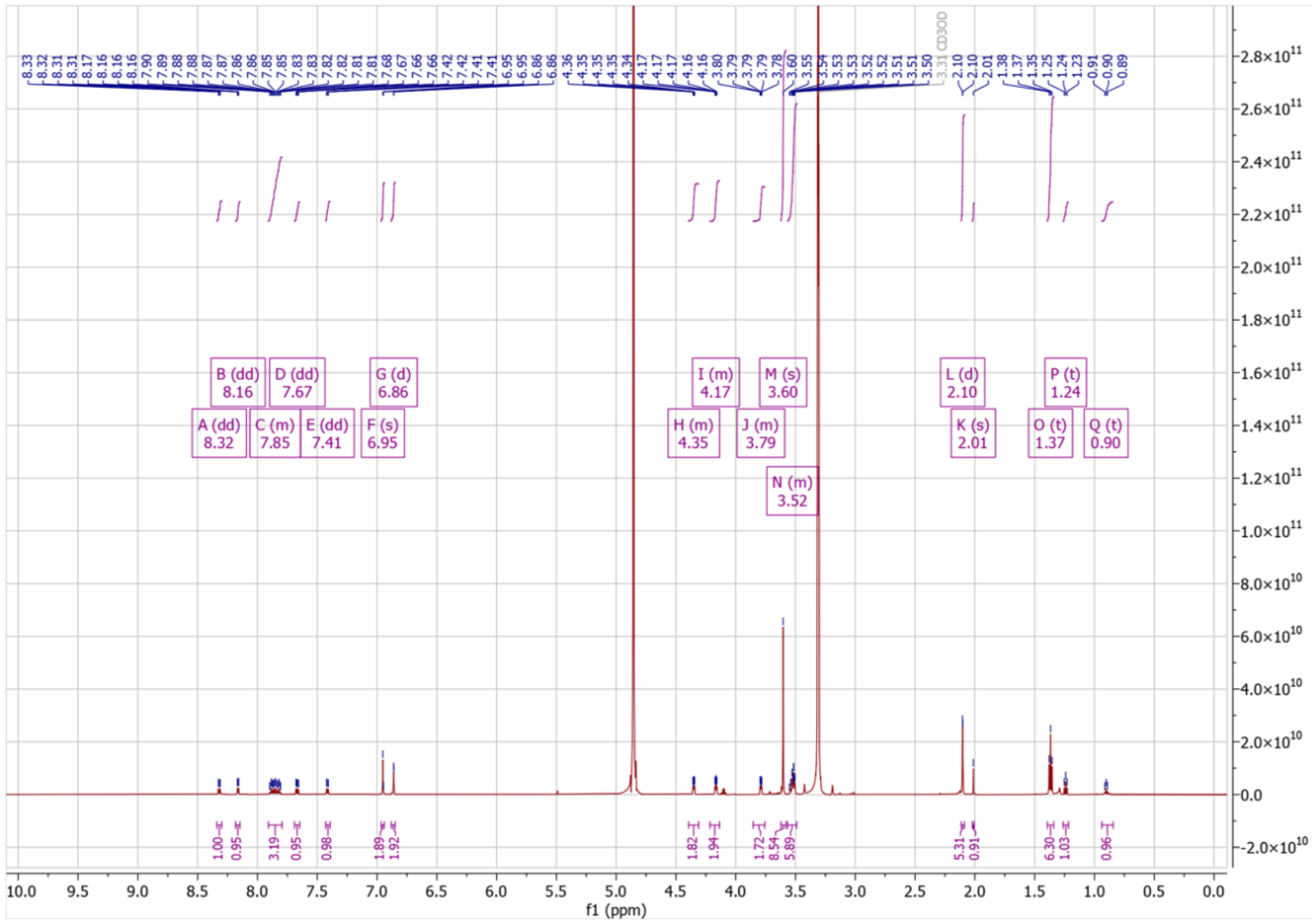

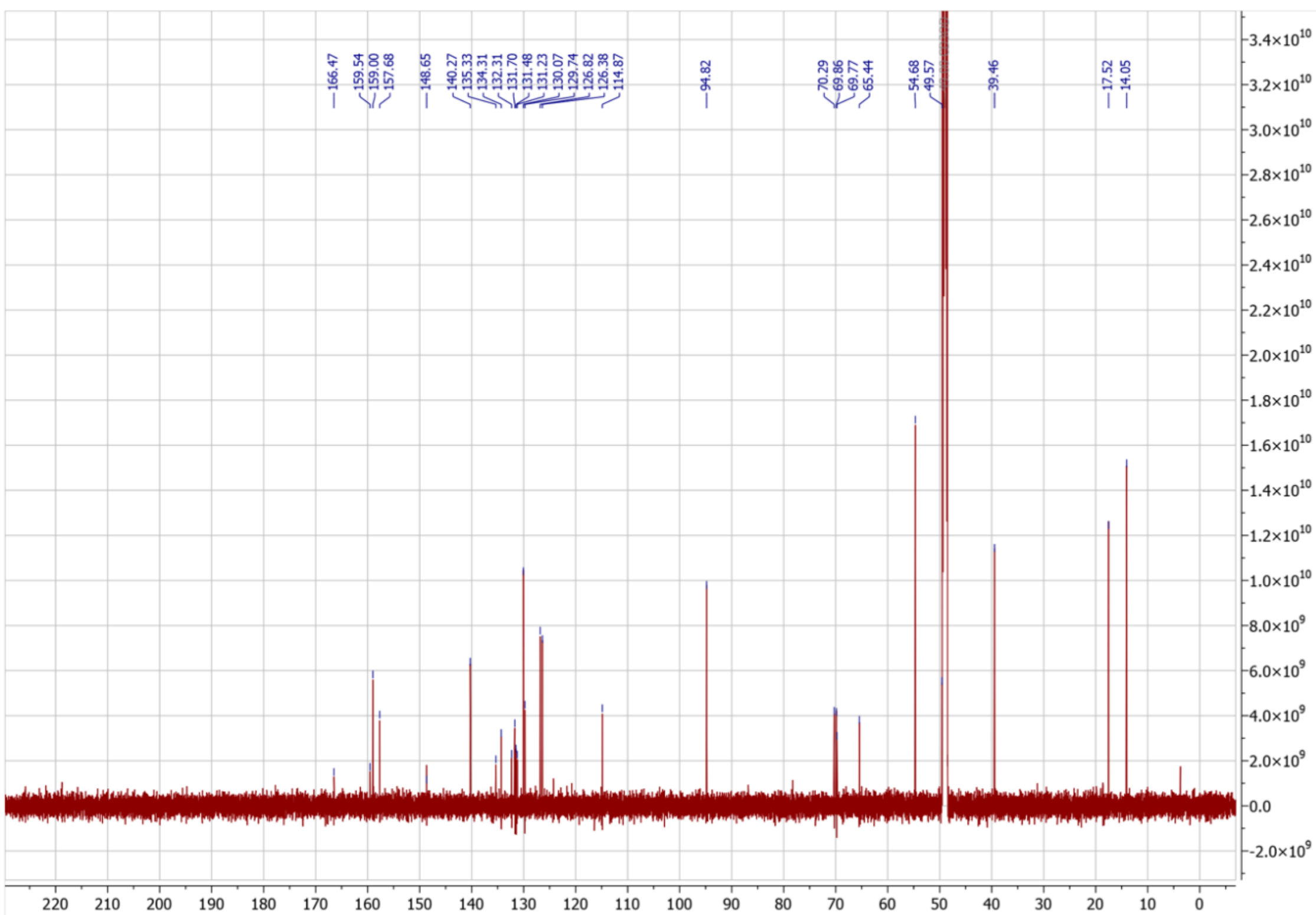

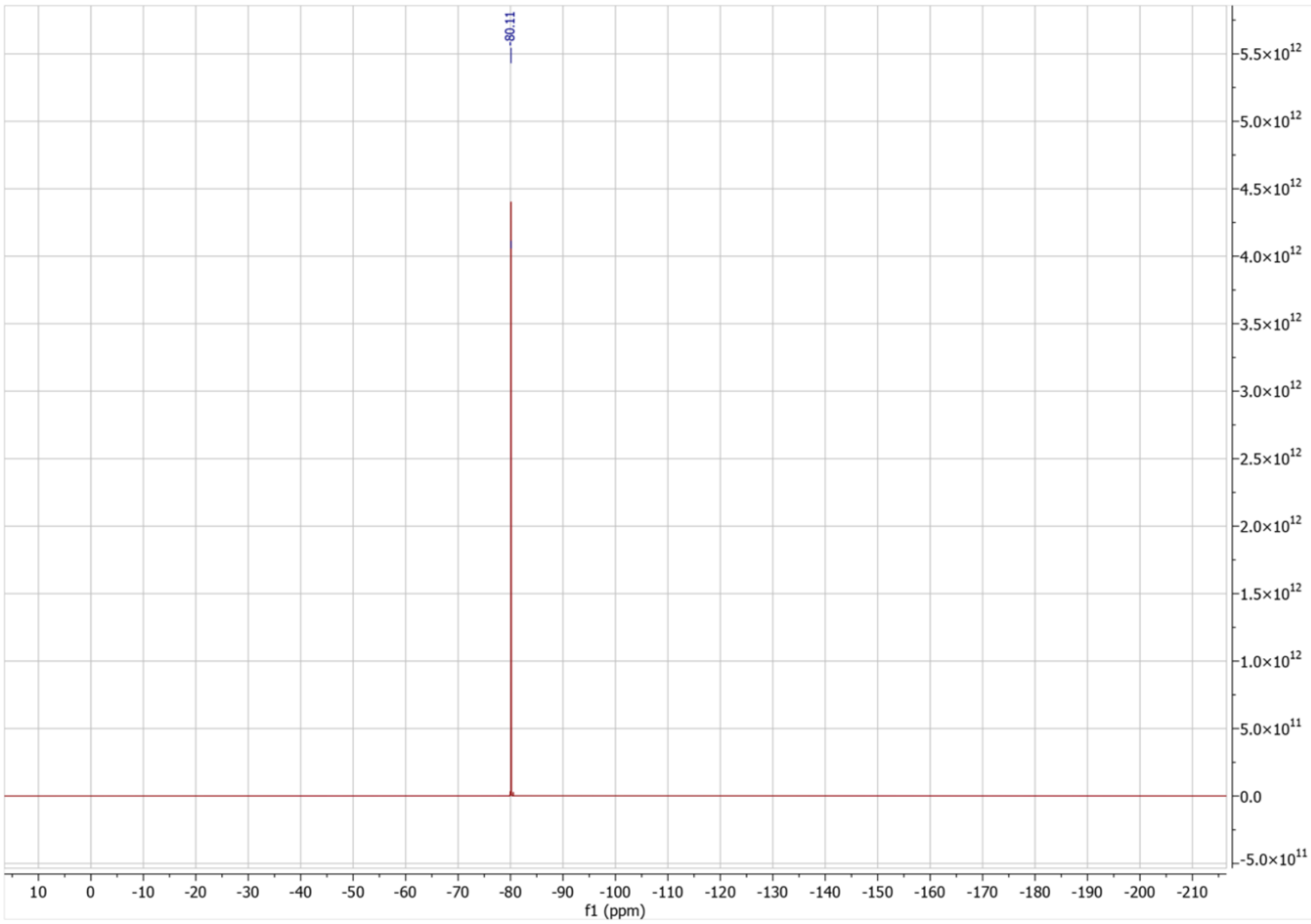
-80.11
f1 (ppm)

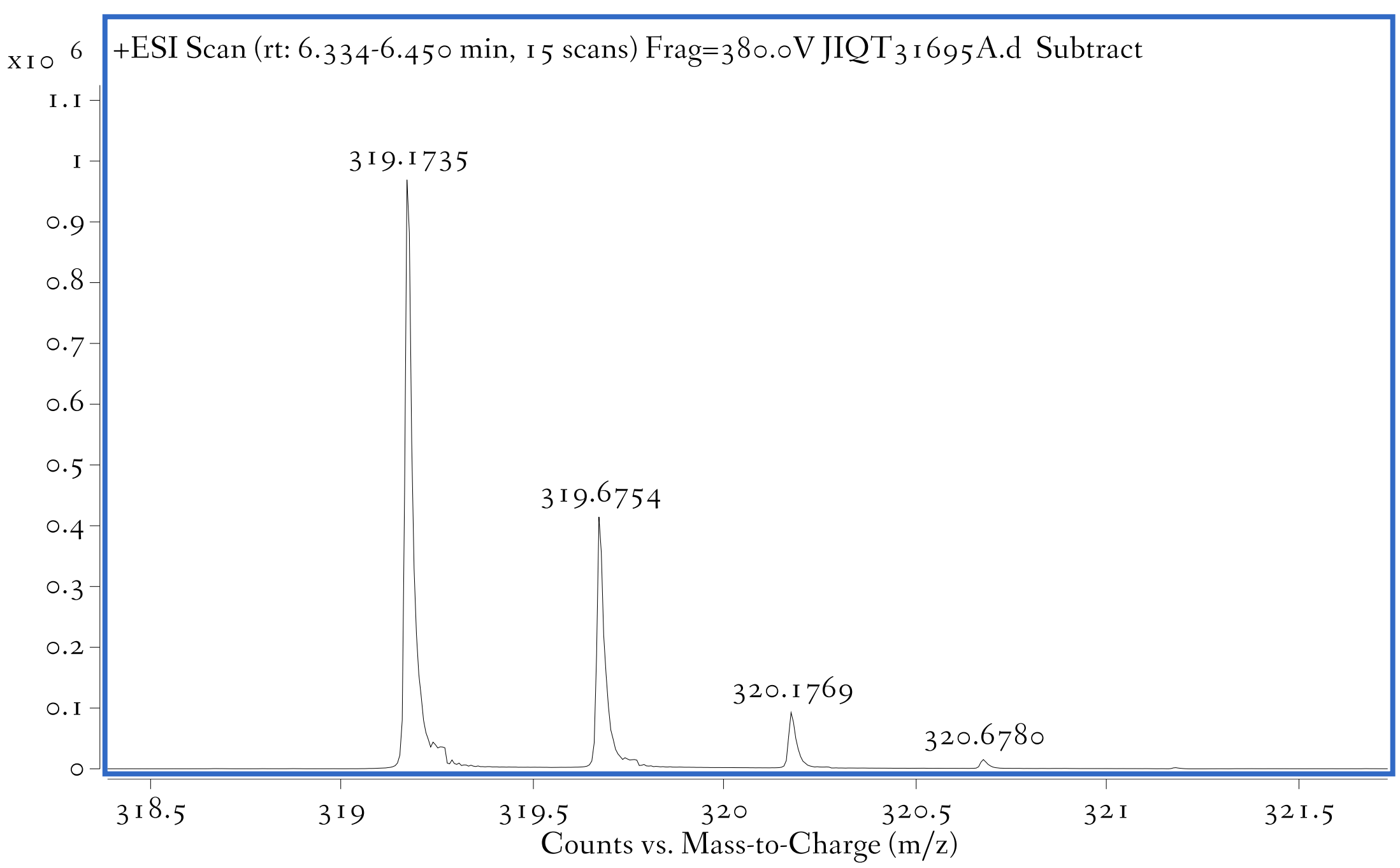
+ESI Scan (rt: 6.334-6.450 min, 15 scans) Frag=380.0V JIQT31695A.d Subtract
x10 6
319.1735
319.6754
320.1769
320.6780
Counts vs. Mass-to-Charge (m/z)
